# Patrilocality and hunter-gatherer-related ancestry of populations in East-Central Europe during the Middle Bronze Age

**DOI:** 10.1038/s41467-023-40072-9

**Published:** 2023-08-01

**Authors:** Maciej Chyleński, Przemysław Makarowicz, Anna Juras, Maja Krzewińska, Łukasz Pospieszny, Edvard Ehler, Agnieszka Breszka, Jacek Górski, Halina Taras, Anita Szczepanek, Marta Polańska, Piotr Włodarczak, Anna Lasota-Kuś, Irena Wójcik, Jan Romaniszyn, Marzena Szmyt, Aleksander Kośko, Marcin Ignaczak, Sylwester Sadowski, Andrzej Matoga, Anna Grossman, Vasyl Ilchyshyn, Maryna O. Yahodinska, Adriana Romańska, Krzysztof Tunia, Marcin Przybyła, Ryszard Grygiel, Krzysztof Szostek, Miroslawa Dabert, Anders Götherström, Mattias Jakobsson, Helena Malmström

**Affiliations:** 1 Institute of Human Biology and Evolution, Faculty of Biology, Adam Mickiewicz University in Poznań, Uniwersytetu Poznańskiego 6, 61-614 Poznań, Poland; 2 Faculty of Archaeology, Adam Mickiewicz University in Poznań, Uniwersytetu Poznańskiego 7, 61- 614 Poznań, Poland; 3 Archaeological Research Laboratory, Department of Archaeology and Classical Studies, Stockholm University, Lilla Frescativägen 7, SE-106 91 Stockholm, Sweden; 4 Centre for Palaeogentics, Svante Arrhenius väg 20C, SE-106 91 Stockholm, Sweden; 5 Institute of Archaeology, University of Gdańsk, ul. Bielańska 5, 80-851 Gdańsk, Poland; 6 Department of Anthropology and Archaeology, University of Bristol, 43 Woodland Road, Bristol, BS8 1UU UK; 7 Laboratory of Genomics and Bioinformatics, Institute of Molecular Genetics of the Czech Academy of Sciences, Vídeňská 1083, 142 20 Prague 4, Prague, Czech Republic; 8 Department of History and Cultural Heritage, University of Pope Jan Paweł II, Kanonicza 9, 31-002 Cracow, Poland; 9 Archaeological Museum in Cracow, Senacka 3, 31-002 Cracow, Poland; 10 Institute of Archaeology, Maria Curie-Skłodowska University, M.C.-Skłodowska sq. 4, 20-031 Lublin, Poland; 11 Institute of Archaeology and Ethnology, Polish Academy of Science, Sławkowska 17, 31-016 Cracow, Poland; 12 Department of Material and Spiritual Culture, Lublin Museum, Zamkowa 9, 20-117 Lublin, Poland; 13 Archaeological Museum in Poznań, Wodna 27, 61-781 Poznań, Poland; 14 Muzeum Archeologiczne w Biskupinie, Biskupin 17, 88-410 Gąsawa, Poland; 15 Zaliztsi Museum of Local Lore, Schevchenka 51, Zalizhtsi, 47243 Ternopil reg, Ukraine; 16 Ternopil Regional Center for Protection and Research of Cultural Heritage Sites, Kyyivsʹka 3а, 46016 Ternopil, Ukraine; 17 Wojewódzki Urząd Ochrony Zabytków, Gołębia 2, 61-840 Poznań, Poland; 18 Archaeological company “Dolmen Marcin Przybyła, Michał Podsiadło s.c.”, Serkowskiego Sq. 8/3, 30-512 Cracow, Poland; 19 Museum of Archaeology and Ethnography in Łódź, Plac Wolności 14, 91-415 Łódź, Poland; 20 Institute of Biological Sciences, Cardinal Stefan Wyszynski University in Warsaw, Wóycickiego 1/3, 01-938 Warsaw, Poland; 21 Molecular Biology Techniques Laboratory, Faculty of Biology, Adam Mickiewicz University in Poznań, Uniwersytetu Poznańskiego 6, 61-614 Poznań, Poland; 22 Human Evolution, Department of Organismal Biology, Uppsala University, Norbyvägen 18C, SE-752 36 Uppsala, Sweden; 23 Centre for Anthropological Research, University of Johannesburg, Auckland Park, 2006 Johannesburg, South Africa; 24 SciLifeLab, Stockholm and Uppsala, Sweden

**Keywords:** Population genetics, Archaeology

## Abstract

The demographic history of East-Central Europe after the Neolithic period remains poorly explored, despite this region being on the confluence of various ecological zones and cultural entities. Here, the descendants of societies associated with steppe pastoralists form Early Bronze Age were followed by Middle Bronze Age populations displaying unique characteristics. Particularly, the predominance of collective burials, the scale of which, was previously seen only in the Neolithic. The extent to which this re-emergence of older traditions is a result of genetic shift or social changes in the MBA is a subject of debate. Here by analysing 91 newly generated genomes from Bronze Age individuals from present Poland and Ukraine, we discovered that Middle Bronze Age populations were formed by an additional admixture event involving a population with relatively high proportions of genetic component associated with European hunter-gatherers and that their social structure was based on, primarily patrilocal, multigenerational kin-groups.

## Abstract (web summary)

By analysing 91 Bronze Age genomes from East-Central Europe, the authors discovered that Middle Bronze Age populations were formed by an admixture event involving hunter-gatherers and that the social structure of resulting population was primarily patrilocal.

## Introduction

Currently, it is well established that the European gene pool has been shaped by several major demographic events, including the postglacial spread of hunter-gatherers^1^; subsequent migrations of early farmers, which marked the beginning of the Neolithic in Europe^2,3^; and the later arrival of Pontic-Caspian steppe pastoralists^4–6^. However, there is still extensive debate surrounding the scale and exact nature of these demographic events and how they affected the genetic makeup of different regions across Europe^7^.

East-Central Europe, in particular, is a region often on the frontier of these events, resulting in a mosaic of genetically distinct populations associated with a variety of cultural entities. By the turn of the Bronze Age, this region was dominated by populations associated with the Corded Ware Culture (CWC) and Bell Beaker Culture (BBC) and characterised by high levels of steppe ancestry^4–6,8^. Descendants of steppe pastoralists are thought to have replaced and admixed with the late Neolithic populations, which were characterised by large amounts of Anatolian farmer ancestry with additional low to medium levels of hunter-gatherer ancestry^9,10^. However, the Funnel Beaker Culture (FBC) and Globular Amphora Culture (GAC), major entities predating the arrival of steppe pastoralists associated with the later Neolithic in the region, are thought to have been long lasting, with some of their local variants continuing well into the Early Bronze Age (up to 2000 BCE, in the case of the GAC)^11^. As no ancient DNA (aDNA) data are available from individuals associated with later populations, their genetic makeup can only be inferred from their 3rd millennium-BC counterparts. The northern and eastern parts of East-Central Europe followed slightly different trajectories, with populations associated with the sub-Neolithic forest zone^12,13^, characterised by high levels of hunter-gatherer ancestry (and, to a large extent, lifestyle) throughout the Neolithic. In these regions, Anatolian farmer ancestry was introduced along with steppe ancestry during the onset of the Bronze Age^14,15^. Similar patterns have been observed in other regions surrounding the Baltic Sea, such as southern Scandinavia and Gotland, where hunter-gatherer individuals (both in terms of lifestyle and genetic composition) of the Pitted Ware Culture (PWC) coexisted in close proximity (but without significant gene flow) with the FBC in the 4th millennium BCE and the Battle Axe Culture (BAC), the Scandinavian variant of the CWC, in the first half of the 3rd millennium BCE^16,17^.

The cultural landscape of Early Bronze Age (EBA) in East-Central Europe (2400–1800 BCE) is widely believed to be a direct continuation of processes that started during the onset of the epoch. For example, the cultural entities present in the region, such as those associated with the Mierzanowice, Iwno and Strzyżów archaeological cultures (MC, IC and SC, respectively)^18–20^^,^ are largely seen as continuations of groups associated with the CWC and BBC^21–23^. In addition, steppe^24,25^ or the northern forest zone^26^ cultures have been suggested to have influenced, to some extent, the SC.

**Fig. 1:**
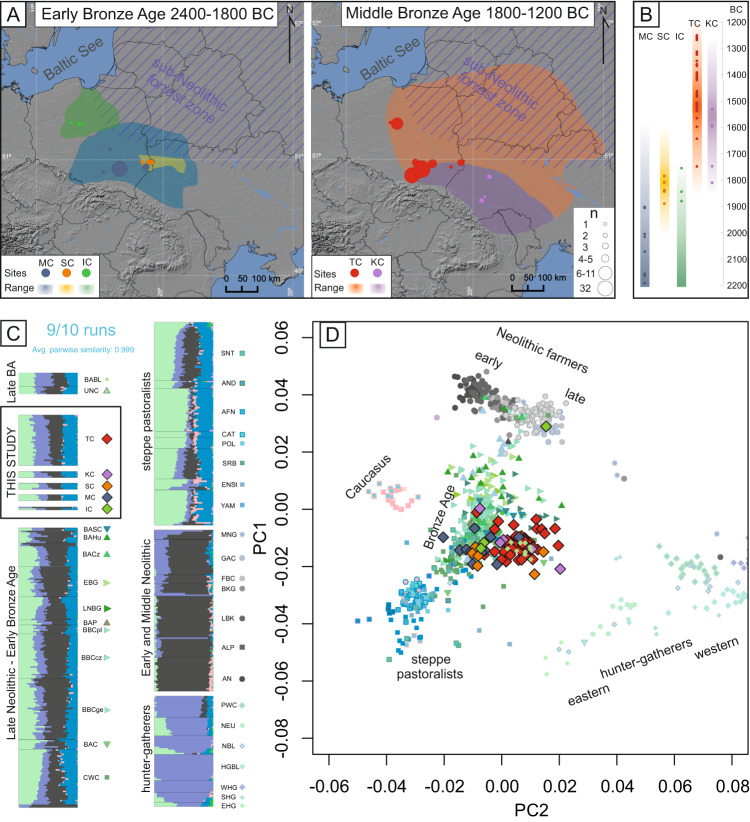
The geographical and temporal context and genetic affinities of the analysed Bronze Age individuals. **A** Maps showing the locations of samples published in this study and the geographical range of their associated cultural entities; the size of the marker corresponds to the number of samples from each site. The map was created using QGIS 2.12.2^49^ and basemap from NOAA National Geophysical Data Center. 2009: ETOPO1 1 Arc-Minute Global Relief Model. NOAA National Centers for Environmental Information. Accessed 2013. **B** The age of the newly generated genomes (calculated as an average of 2*σ* BCE dates) corresponding to the temporal range of the archaeological cultures they are associated with. **C** The results of unsupervised admixture analysis (K = 7) on the selected populations. **D** PCA plot of ancient individuals projected onto contemporary individuals from West Eurasia from the Human Origins reference panel (not shown). The symbols in both the PCA and admixture analysis correspond to individuals associated with the following cultures: IC - Iwno Culture, KC – Komarów Culture, MC – Mierzanowice Culture, SC – Strzyżów Culture, TC – Trzciniec Culture (from this study and reference populations), AFN – Afanasievo Culture, ALP – Alföld Linear Pottery Culture, AN – Anatolian Neolithic, AND – Andronovo Culture, BABL – Bronze Age Baltic, BAC – Battle Axe Culture, BACz Bronze Age Czechia, BAHU – Bronze Age Hungary, BAP – Bronze Age Poland, BASC – Bronze Age Scandinavia, BBC – Bell Beaker Culture (Poland, Czechia and Germany, respectively), BKG – Brześć Kujawski Group, CAT – Catacomb Culture, CWC – Corded Ware Culture, EBG – Early Bronze Age Germany, EHG – Eastern Hunter Gatherers, ENSt – Eneolithic Steppe, FBC – Funnel Beaker Culture, GAC – Globular Amphora Culture, HGBL – Hunter Gatherer Baltic, LBK – Linear Pottery Culture, LNBG – Late Neolithic/Bronze Age Germany, MNG – Middle Neolithic Germany, NBL – Neolithic Baltic, NEU – Neolithic Ukraine, POL – Poltavka Culture, PWC – Pitted Ware Culture, SHG – Scandinavian Hunter Gatherers, SNT – Sintashta Culture, SRB – Srubnaya Culture, UNC – Únětice Culture, WHG – Western Hunter Gatherers, YAM – Yamnaya Culture.

The Middle Bronze Age (MBA) in the region (1800–1200 BCE) was in turn dominated by the Trzciniec Cultural Circle (TCC). This cultural phenomenon extended from the Oder River drainage basin to the Desna and Seym River basins (ca. 1200 km) and from the Baltic seashore to the Prut basin (ca. 750 km), exhibiting several territorial variants^27^. This study focuses on MBA individuals associated with two of these variants (Fig. 1A): the Trzciniec Culture (TC), which occupied the lands belonging to modern-day Poland and central-western Ukraine, and the Komarów Culture (KC), found in modern-day southwestern Ukraine and neighbouring parts of Romania and Moldova^27^. These MBA cultures retained many cultural aspects of their EBA counterparts, such as styles of pottery and bronze artefacts as well as funeral practices including under-barrow graves and cremation^27^. This cultural similarity have been shown to coincide with genetic continuity between CWC and both EBA and MBA populations as seen in mitochondrial genome data^28^. However, some elements of the TCC are unique to the EBA or MBA, particularly the predominance and scale of collective burials. Collective burials of multiple individuals were also prevalent among Middle and Late Neolithic populations in Central and East-Central Europe associated with local variants of the FBC or GAC^29,30^. Recent studies have shown that these Neolithic collective burials often contain remains of multiple individuals who belonged to, in most cases, patrilocal kin groups^31–34^. The biological relatedness among individuals found in these MBA collective burials associated with the TCC, remains unexplored. The presence or absence of kinship among these populations would, however, greatly increase our understanding of the social organization and structure of these societies. The re-emergence of collective burials is the subject of another debate concerning whether this predominant burial custom in the TCC was a result of genetic shifts or social changes within the Bronze Age populations^30^.

This study, explores genetic affinities between individuals from various EBA and MBA cultures and their genetic relations to populations of preceding cultural complexes as well as possible kinship structures within MBA societies. To achieve this we conducted population genetic analyses using 91 newly generated genomes from Bronze Age individuals associated with EBA and MBA cultures from modern-day southern and south-eastern Poland and western Ukraine.

## Results and discussion

Out of the 175 Bronze Age individuals screened, 92 produced enough data (>0.018 genome coverage) to be retained for further analysis and/or deeper sequencing. An additional 100 libraries, including 37 uracil-DNA-glycosylase (UDG)-treated libraries, were created and sequenced for the selected individuals. With the exception of two libraries from two different individuals, all libraries displayed characteristic post-mortem damage at the 5′ and 3′ ends of aDNA fragments and low contamination levels, as estimated by mitochondrial sequences and X chromosome sequences in males, where sufficient coverage was obtained (Supplementary Data 1 and Supplementary Data 2). Only one library for individual poz751, which displayed very low levels of post-mortem damage, and one of two libraries for individual poz664, in which high levels of mitochondrial DNA (mtDNA) contamination were detected, were excluded from further analysis. Therefore, the final dataset used for the kinship and population genetic analyses consisted of 91 individuals with a median genome coverage of 0.2× (ranging from 0.019× to 2.29×), representing the IC (*n* = 3), MC (*n* = 15), SC (*n* = 6), TC (*n* = 62) and KC (*n* = 5) cultures.

### Genetic affinities of Early Bronze Age populations from East-Central Europe indicate the continuation of processes initiated in the onset of the epoch

The majority of the EBA individuals in this study (2200–1850 BCE) associated with the MC, IC and SC are genetically similar to their direct cultural predecessors (such as the BBC and CWC), as indicated by the principal component analysis (PCA) plot (Fig. 1D). The results of unsupervised admixture analysis (K = 7) of the selected populations (Fig. 1C) support these relationships as they indicate similar levels of admixture components (Fig. 1C). These findings are consistent with the general archaeological consensus and previous analyses of mitochondrial data^28^ Although the IC is believed to have the greatest cultural similarity to groups associated with the BBC, the one individual analysed here (poz929) exhibited closer affinity with individuals associated with various CWC groups rather than the BBC populations according to the f3 and *D* statistics (Supplementary Data 7, Supplementary Data 12 and Supplementary Fig. 2). A similar trend was observed in the case of individuals attributed to the MC, which displayed closer affinity to CWC individuals from Estonia over other EBA groups. However, the division between the BBC and CWC (and their definition as independent cultural entities associated with distinct populations) is contested^7^, and the majority of the above D statistics have low *Z* scores. Further studies are needed to fully understand the regional, cultural and genetic complexity of Central and Eastern Europe after the arrival of steppe pastoralists.

One IC-associated male from Łojewo (poz502) deviated from the general pattern, as he was genetically closest to the Middle and Late Neolithic populations, which occupied the same space on the PCA plot and displayed similar admixture proportions. The results of *f3* statistics indicate that the population sharing the most genetic drift with this individual was the GAC, followed closely by the FBC (Supplementary Data 7). Such seemingly Neolithic individuals have occasionally been observed in populations postdating the arrival of steppe pastoralists and have been hypothesised to be foreigners who were incorporated into Bronze Age societies from isolated populations that retained a Middle Neolithic genetic makeup up to the end of the 3rd millennium BCE^35^. If this interpretation is applied to poz502, radiocarbon dated to the border between the EBA and MBA (2008-1750 BCE), it might indicate that such isolated populations lasted far longer than previously reported. This hypothesis is supported by the archaeological record, which shows that some Neolithic cultures, most notably the GAC, lasted well into the Bronze Age^21^.

Similar to poz502, two of the SC-associated males (poz794 and poz758) differed genetically from other EBA individuals. These two males were closer to the hunter-gatherer space in the PCA plot (Fig. 1D) and showed an increased proportion of genetic components that were maximised in various European hunter-gatherer populations in the admixture analysis (Fig. 1C). However, direct radiocarbon dating for one of these individuals (poz794, 1921–1697 BCE) as well as their genetic similarity to the MBA populations analysed in this study indicate that these two males should be discussed as a part of the genetic shifts observed in the MBA. Notably, the SC is generally thought to be a regional cultural phenomenon with mixed cultural traits, leading to frequent dispute over the association of individual burials or sites with this culture^24–26^. Therefore, the definition and associations of the SC with any genetically distinct population warrants further exploration targeting a broader selection of individuals attributed to this culture.

### Increase in hunter-gatherer ancestry in East-Central Europe in the Middle Bronze Age

The MBA individuals analysed here were dated to a range between 1750 and 1200 BCE and were associated with the TCC, representing both the TC and KC. The majority of these individuals clustered together in PCA space and shared similar admixture proportions (Fig. 1C and D). This apparent genetic relation is further highlighted by *f3* and *D* statistics, which indicate that when analysed separately, KC and TC individuals do not, in majority of cases, display any statistically significant closer genetic affinity to either of the two populations (Supplementary Data 7 and Supplementary Data 12, Supplementary Figs. 3C and 4B). These results are in accordance with the archaeological interpretation that questions the separation of the TC and KC, arguing in favour of treating them as regional variants of the same phenomenon^27,36^.

Interestingly compared to EBA populations, the MBA individuals were closer in the PCA space to various hunter-gatherer populations from Europe (Fig. 1D), something that previously was not detected in analyses of mitochondrial genome data alone^28^. Moreover, admixture analysis indicated elevated amounts of genetic components maximised in hunter-gatherers (Fig. 1C). This suggests an additional admixture event at the beginning of the MBA involving a population with relatively high proportions of this genetic component. However, there were notable deviations to this trend, with three individuals associated with TC from Pielgrzymowice site and poz643, a relatively early KC male from Beremiany, clustering closer to EBA populations in PCA space and displaying the lowest levels of shared genetic drift with both TC and hunter-gatherer populations (Supplementary Data 7). When using qpAdm to test for possible two-way admixture models that resulted in the formation of MBA populations, several models were determined to be plausible (Supplementary Data 13) with the highest *p* value (*p* = 0.21) obtained for pair consisting of IC and Neolithic Baltic hunter-gatherers (NBL). Similarly high pvalues were found for other pairs including IC and other hunter-gatherer populations: Western Hunter Gatherers (WHG), PWC hunter-gatherers from Gotland, hunter-gatherer buried in BKG context (BKGout) and hunter-gatherer populations predating the NBL (HGBL) (*p* = 0.207, 0.204, 0.164, 0.160 respectively). These results are in accordance with the archaeological hypothesis that the IC greatly contributed to the emergence of the TC^27^. However, this finding should be interpreted with caution, as only one individual associated with the IC had sufficient coverage for inclusion in qpAdm. High fit values were also obtained for pair of populations, containing individuals from modern day Estonia associated with the CWC, as the EBA predecessor and PWC, as the source of hunter-gatherer ancestry (*p* = 0.11).

The process of admixture was likely more complex, as indicated by the three-way admixture model with the addition of a Neolithic population. These models yielded even better fits than the two-way models. Multiple plausible scenarios were found, all displaying high fit values (*p* > 0.9) including CWCes in addition to various hunter-gatherer and Neolithic populations (Supplementary Data 14). The more discriminatory rotating outgroup approach to qpAdm^37^ used for both two- and three- way models helped to narrow down the number of plausible scenarios. After excluding the models containing sources consisting of one individual, only three-way scenarios including CWCes and either GAC or FBC as Neolithic and NBL or HGBL as hunter-gatherer population were found to be plausible (Supplementary Data 15 and Supplementary Data 16). Based on geographical and temporal proximity as well as results of D statistics directly comparing the potential ancestry sources for each MBA individual (Supplementary Data 12, Supplementary Fig. 3) we find CWCes, NBL and GAC to be the best proxies for populations involved in the admixture process. However high fit values and close genetic affinity to the individual with hunter-gatherer ancestry buried in BKG context, indicate that further studies might help to better define the populations involved in the process.

The most likely hypothesis is that these admixed MBA populations originated in the confluence of the sub-Neolithic forest zone, associated with populations with dominant WHG ancestry^14,15^ as well as post-CWC groups characterised by a large proportion of steppe ancestry. The sub-Neolithic forest zone” is a broad term that includes various archaeological cultures from north-eastern Europe, characterised by long-lasting preservation of a predominantly hunter-gatherer lifestyle, and the incorporation of cultural elements of Neolithic and Bronze Age origin^38^. These populations remained genetically distinct from the Neolithic and post-Neolithic populations, although they maintained some level of long lasting cultural and economic exchange^14^ It is possible that this lead to some degree of gene flow between those populations, similar to the one observed in the case of PWC in Gotland^6,16^ followed by subsequent contacts with EBA descendants of steppe pastoralists. Moreover, the TCC and sub-Neolithic forest zone exhibited similar cultural traits, mostly in the form of pottery and technologies^12,13,39–41^. These similarities have often been interpreted as signs of primarily cultural exchange. Our results, showing an increase in WHG ancestry during the MBA, indicate that at least some level of admixture occurred during these interactions.

Notably, the two EBA individuals (poz794 and poz758) associated with the SC, that displayed closer genetic affinity to the MBA populations both came from the south-eastern part of modern-day Poland (Supplementary Text); of these two, the individual with a direct date (poz794) predated the MBA samples analysed here. This observation suggests that the contact zone described above is not the only place where admixture took place and/or that the process was more geographically diffused. Either is plausible, given the range and duration of exchange networks seen in the EBA^40^. Individual poz794 might even signal the beginning of the observed gene flow, which would date it to approximately 1800 BCE.

The SC is usually seen as a continuation of CWC traditions with additional elements from steppe cultures such as the Catacomb Culture^24,25^. However, the genetic shift toward an increase in WHG ancestry cannot be explained by additional migration from the steppe, a notion that we previously proposed based solely on mitochondrial data^28^. This idea is not supported by any indication of increase of steppe ancestry as calculated by three-way qpAdm modelling including WHG, AN and YAM as best proxies of major ancestries in European gene pool (Supplementary Data 17, Supplementary Fig. 4A). Moreover, two-way qpAdm models that explored the scenarios resulting in the emergence of the MBA populations, including EBA individuals and hunter-gatherer populations, yielded higher probabilities than models including additional steppe populations such as Andronovo, Afanasievo, Sintashta, Poltavka, Karasuk, or Srubnaya (Supplementary Data 13).

**Fig. 2:**
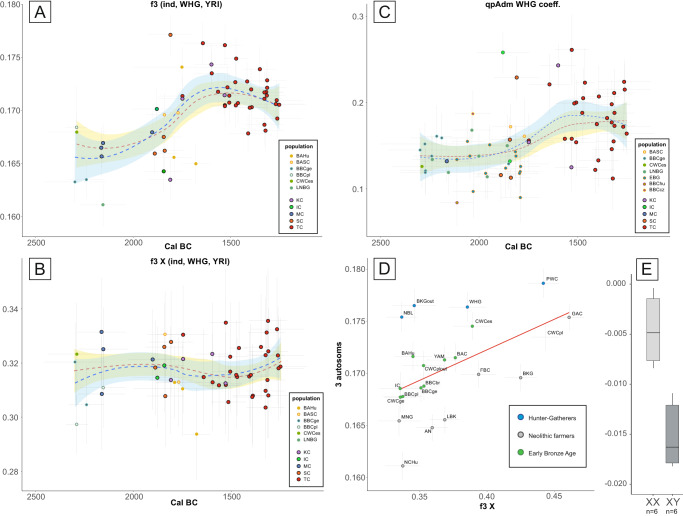
The hunter-gatherer ancestry in the Middle Bronze Age populations from East-Central Europe. **A** The shared genetic drift between the newly published individuals and WHG hunter-gathers was estimated with the use of the *f3*-statistic, shown separately for autosomal and (**B**) X-chromosome data. **C** the WHG ancestry estimated for new (outlined in black) and reference individuals from the Final Neolithic to the Middle Bronze Age. The degree of ancestry was estimated from three-way admixture models including the WHG, AN and YAM, the points represent coefficient for WHG ancestry calculated using qpAdm (only values with *p* value for nested models <0.05). Error bars in (**A**–**C**) correspond to one standard error for the *f3*-statistics or qpAdm values (vertical) and 2*σ* for the dates (horizontal). The fit lines in (**A**–**C**) display smoothed conditional means for all individual (blue) and after removal of outliers (red) with corresponding 95% confidence intervals (light blue and yellow respectively). **D** Outgroup f3 statistics values of form f3_Xchr(YRI, TC, popX) and f3_Autosomes(YRI, TC, popX) plotted against each other with error bars representing one standard deviation for each value, red line represents linear regression inserted for visualisation purpose. **E** The patrilocal character of Żerniki Górne cemetery as shown by the difference in the *D*-statistic in the form *D*(YRI, Żerniki individual; Żerniki, TC) for the two sexes. The boxplots show the median (middle horizontal line), interquartile range (25th and 75th percentile) indicated with lower (25th percentile) and upper (75th percentile) hinges of the box, and whiskers extending to the lowest (highest) value that is within 1.5 times the interquartile range of the upper (lower) hinge. The labels in all panels are as follows: IC – Iwno Culture, KC – Komarów Culture, MC – Mierzanowice Culture, SC – Strzyżów Culture, TC – Trzciniec Culture, AN – Anatolia Neolithic, BAC – Battle Axe Culture, BAHu – Bronze Age Hungary, BAP – Bronze Age Poland, BASC – Bronze Age Scandinavia, BBC – Bell Beaker Culture, BKG – Brzesc Kujawski Group, CWC – Corded Ware Culture, EBG – Early Bronze Age Germany, FBC – Funnel Beaker Culture, GAC – Globular Amphora Culture, LNBG – Late Neolithic/Bronze Age Germany, MNG Middle Neolithic Germany, NBL Neolithic Baltic, NCHu Neolithic/Chalcolithic Hungary.

The process of admixture, which began around 1800 BCE, appears to have been a continuous rather than a result of a single migratory event, as evidenced by the presence of individuals with very high or very low proportions of hunter-gatherer ancestry throughout the whole temporal range of MBA samples analysed here (Fig. 2C, Supplementary Data 17). However, gene flow was likely more extensive in the beginning, as both shared genetic drift (as identified by *f3* statistics) and admixture proportions (calculated with qpAdm) show that the proportion of hunter-gatherer ancestry decreased slightly over time (Fig. 2A and Fig. 2C). The results of this event must have been long lasting, as Late Bronze Age individuals from modern-day Latvia and Lithuania^14^ retain the same genetic composition as our MBA individual despite living nearly half a century later and were found, based on *f3* statistics, to have the closest genetic affinity to the MBA individuals presented here out of all Bronze Age populations (Supplementary Data 7 and Supplementary Fig. 3C).

Furthermore, several lines of reasoning support the idea that this admixture event was dominated by males originating in a population characterised by a high level of hunter-gatherer ancestry. First, as shown by our direct kinship analyses below, the resulting population was primarily patrilocal. Second, the MBA composition of Y-DNA haplogroups differed significantly from the predating populations, as dominance of I2a1a and I2a1b haplogroups was previously seen only sporadically in various hunter-gatherer populations, including two Narva Culture individuals^14^ and interestingly in high frequency, although for different sub-haplogroups, in GAC collective burials^10,34,42^. The I2a1 haplogroups were found in 75% of TC-associated MBA individuals, even after selecting only one individual from each detected kin group. Moreover, this shift was not apparent when looking at the mitochondrial haplogroups^28^. Finally, direct analysis of genetic distances in X-chromosome data, as determined by *f3* statistics using the approach suggested by Saag et al.^15^ showed that on autosomes TC was relatively more similar to hunter-gatherer populations than on X chromosome (Fig. 2D, Supplementary Data 5 and Supplementary Data 6). In addition, when looking at the temporal changes in X-based f3 values we did not observe increased amounts of genetic drift shared with WHG individuals between 1800 and 1500 BCE, as seen in the autosomal data (Fig. 2B, Supplementary Data 8).

The exact trajectory of events leading to the genetic shift in the MBA cannot be reconstructed with current knowledge. The Eastern Baltic hunter-gatherer populations were associated with multiple archaeological cultures that engaged in direct contact with Neolithic farmers for millennia. It cannot be excluded that at some points of this coexistence, migratory events occurred, leading to the emergence of admixed populations that, in turn, later mixed with steppe pastoralists or their Central European descendants, resulting in the formation of MBA populations analysed here. The lack of more diverse genetic data from East-Central Europe prevents us from pinpointing the exact populations that took part in this admixture. As the archaeological record shows that contact between culturally distinct groups of farmers and hunter-gatherers were long lasting, leading to substantial cultural changes^38^, it is possible that the practices of collective burials and patrilocal residence were some of those changes. This could be reflected in the high frequency of I2a1b Y haplogroup in some collective burials associated with middle Neolithic GAC culture^10,34,42^.The observed changes could have resulted from several processes involving multiple populations; our observations represent the sum of those processes.

### Paternal kinship structure among the Bronze Age collective burials

**Fig. 3:**
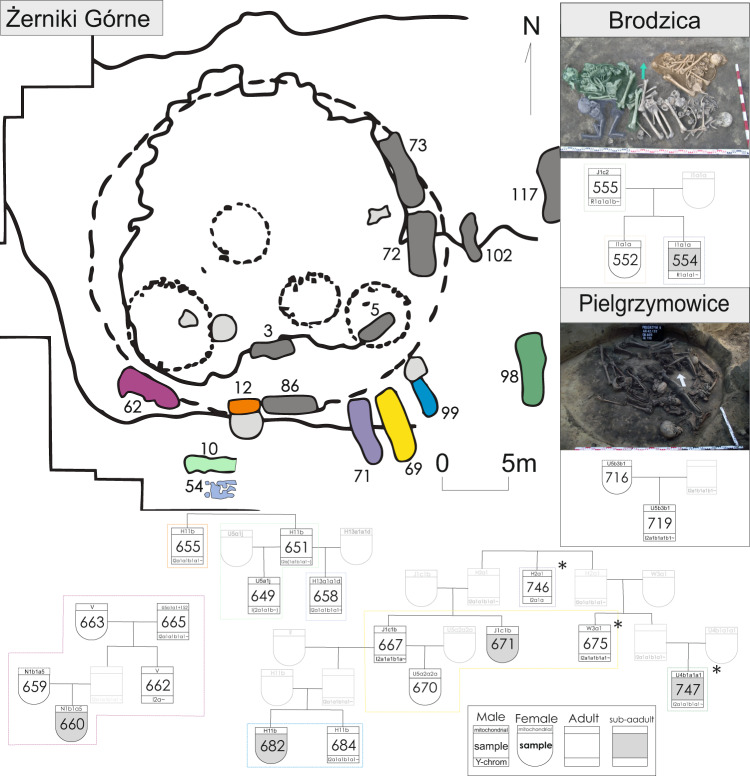
The kinship structure of the Middle Bronze Age populations from East-Central Europe. The proposed pedigrees of detected kin groups from Bronze Age cemeteries associated with the Trzciniec culture, asterisk (*) marks instances where more than one interpretation of the detected kinship is possible. The colours correspond to either the collective burials on the plan of the site in which the individuals were interred (in the case of Żerniki Górne) or the skeletal remains within collective burials. The burial photographs were reprinted with permission from Wiley from Juras, A. et al. Mitochondrial genomes from Bronze Age Poland reveal genetic continuity from the Late Neolithic and additional genetic affinities with the steppe populations. Am. J. Phys. Anthropol. 172, 176–188 (2020); © 2020 Wiley Periodicals, Inc All Rights Reserved.

The Trzciniec Cultural Circle stands out from the other Bronze Age populations in East-Central Europe due to the high number of TC and KC-associated individuals buried in collective burials. In this study, we analysed 62 individuals from 12 archaeological sites, 52 of which were buried within structures containing remains of at least two people. In addition, as in the case of two individuals from the Dacharzów site, single graves were often in close proximity to collective burials; for example, beneath the burial mounds constructed over them^30^. Our data clearly show that MBA collective burials associated with the TCC contained numerous genetically related individuals, with multiple first- and second-degree kinships found within those structures (Supplementary Fig. 5). The largest number of close relationships was detected among individuals from the Żerniki Górne cemetery, which displayed the best overall aDNA preservation. Out of 28 analysed individuals interred in 9 structures, 17 individuals were found to belong to kin groups that, in some cases, had reconstructed pedigrees spanning at least 4 generations (Fig. 3). Interestingly, direct genetic kinship was also found between individuals buried in different, although adjacent, burial chambers. This shows that not only did the graves themselves represent kin groups within the population, but also that the spatial relations of graves within the cemetery represented kin relations. The prevalence of close kinship among adult male descendants compared to adult females suggests that patrilocality was the dominant marriage arrangement. This notion is further supported by higher mitochondrial diversity compared to Y-DNA diversity and larger average genetic distances between females than males. The latter is supported by *D* statistics that showed that males displayed greater tendency to form a clade with other Żerniki Górne individuals over the general TCC-associated population (Fig. 2E, Supplementary Data 10).

However, not all analysed individuals within the same collective grave were genetically related. This finding could reflect the inability to sample all individuals or the inability to characterise them due to insufficient DNA preservation. Moreover, those without detectable kinship in the cemetery were mostly females (2 males and 9 females without detectable kin at Żerniki Górne), which further supports the notion of patrilocality. In some cases, such as Pielgrzymowice grave no. 9, the burial pit/chamber was used for an extended period^43^ and possibly spanning multiple generations; therefore, detecting multiple first- and second-degree kinships was less likely. That said, two out of five analysed individuals shared first-degree kinship and were likely a mother and her adult son. The collective grave at the Brodzica site seems to contain the remains of a nuclear family, and a sufficient amount of data was available for three out of the four individuals, all of whom were related. These individuals most likely represent a father with his two children. The fourth individual, interpreted as an adult women (although the remains did not yield enough nuclear data for kinship analysis), was found to belong to the same mitochondrial haplogroup as the two children^28^ and thus could potentially be their mother or an additional sibling. The MBA population associated with the TC appeared to be slightly less diverse than its EBA predecessors, according to within-group pairwise *f3* statistics used for diversity estimation. This result was not driven by sites with multiple related individuals, as a similar *f3* distances distribution was found in pairs of individuals from different sites (Supplementary Fig. 4C).

The idea that collective burials represent patrilineal kin groups is in accordance with previous observations of earlier Neolithic populations in Europe^31,33–35^. The prevalence of collective kin-based burials was interrupted by the arrival of steppe pastoralists in the turn of the Neolithic and Bronze Age, leading to the development of more individualised societies, such as those associated with the BBC and CWC. The practice of collective burials never disappeared completely as collective burials occurred throughout the EBA^30^; these graves have been shown to contain the remains of kin groups^44^. We identified one such example at the Hrebenne cemetery and another possibly at the Zubowice site (although the low single-nucleotide polymorphism [SNP] overlap makes this estimation uncertain); both of these collective graves were associated with the MC. The scale of this phenomenon in the TC is, however, much more similar to what was present in Neolithic societies and could be interpreted as a proof of the re-emergence of older traditions.

Patrilocal social structure and male-dominated migrations have been linked with populations and events associated with steppe pastoralists and their descendants^15,45,46^. Hunter-gatherer societies, on the other hand, are commonly thought to have been much more fluid in their postmarital residence preferences, with the majority of modern and historic hunter-gatherer groups displaying bilateral practices^47^. Due to the scarcity of samples, we could not assess the postmarital residence preferred by ancient European hunter-gatherers. On the other hand, recent data obtained on Middle Neolithic farmers from Western and Central Europe show that collective burials in these populations usually comprised related individuals of patrilocal descent^31,33,48^, supporting the notion that those populations or their descendants also played a role in the events that resulted in the genetic shift in the MBA.

The results presented here indicate that EBA people in East-Central Europe buried in the MC, IC and SC contexts were most likely the direct descendants of preceding populations associated with the CWC. In addition, the MBA populations were dominated by patrilocal lineages of apparent hunter-gatherer origin, practising burial customs that, while displaying some elements associated with steppe pastoralists, were most analogous to those practised in the Middle and Late Neolithic cultures, predating the arrival of steppe pastoralists into Central Europe. We conclude that after the introduction of steppe ancestry into European populations, hunter-gatherers and farmers remained genetically distinct in some regions and influenced later demographic and cultural processes, as seen in the genetic composition of MBA populations.

## Methods

### Samples

For all samples. collected for the study, appropriate permits, required by Polish and Ukrainian law, were acquired from the institutions providing the access to the specimen. We sampled teeth and/or the petrous parts of the temporal bone from 176 human skeletal remains for aDNA analyses. The samples originated from the broad range of Bronze Age populations that lived in modern-day Poland and Ukraine. The geographical origin for all the individuals is presented in Fig. 1A created with the use of QGIS 2.12.2^49^. They included TC-, KC-, MC- and IC-associated individuals (Supplementary Data 1). We generated genomic data from 91 individuals, including individuals associated with the TC (*n* = 62), KC (*n* = 5), MC (*n* = 15), SC (*n* = 6) and IC (*n* = 3) (Table S2). Detailed information about each individual sampled can be found in Supplementary Data 1 and Supplementary Information Text.

### Laboratory methods

Sample preparation, DNA extraction and genomic library preparation were conducted in a dedicated aDNA laboratory at Adam Mickiewicz University in Poznan, Poland. The laboratory followed established guidelines for aDNA facilities and utilised UV lamps (254 nm), positive air pressure, and high efficiency particulate air (HEPA)-filtered laminar flow hoods. The laboratories, equipment and nonbiological reagents were regularly decontaminated using bleach and/or DNA-away (ThermoScientific) and UV irradiation.

Prior to DNA extraction, bones and teeth were cleaned with 5% NaOCl, rinsed with sterile water and finally decontaminated with UV irradiation (254-nm wavelength, 12 V) for 60 min on each side in a cross-linker. A Dremel® drill with diamond cutting wheels was used to slice the petrous parts of the temporal bone in half, exposing the structures of the otic capsule. We then drilled these structures and tooth roots to obtain 50–150 mg of bone powder for DNA extraction. DNA was extracted using a silica-spin column protocol^50^ but with the sodium dodecyl sulfate (SDS) in the extraction buffer exchanged for 1 M urea^51^. The final elution was performed in 100 µl of elution buffer (EB) (Qiagen).

Twenty microlitres of DNA extract was converted into single-indexed blunt-end Illumina genomic libraries using P5 and P7 adapters^52,53^, omitting the initial nebulization step due to the fragmented nature of aDNA. In addition, for the selected samples (see Dataset S1 for the library types per sample), UDG-treated libraries were also prepared using UDG and endonuclease VIII (endo VIII) treatment to cut postmortem deaminated sites^54^. From each DNA extract, 1–5 genomic libraries were prepared, and one negative library control was processed for every 8–12 aDNA libraries. Each library was then amplified using five to seven polymerase chain reactions (PCRs) for 12–16 cycles. The amplifications were used to introduce single indices and performed in 25 µl containing a mix of 3 µl of the DNA library template with 12.5 µl of 1 × AmpliTaq Gold® 360 Master Mix (Life Technologies), 0.5 µl of PCR primer IS4 (10 mM) and 0.5 µl of indexing primer (10 µM)^55^. Negative controls were included in both the library preparation and PCR steps. All five to seven PCRs per individual were pooled and purified with AMPure® XP Reagents (Agencourt-Beckman Coulter) according to the manufacturer’s protocol. Pooled libraries were then quantified using the High Sensitivity D1 000 Screen Tape assay on a 2200 TapeStation system (Agilent). The genomic libraries were sequenced at the SciLifeLab SNP & SEQ Technology platform in Uppsala or at the National Genomics Infrastructure (NGI) in Stockholm using (in both locations) the Illumina HiSeq 2500 with v2 paired-end, 125 bp chemistry or HiSeq X Ten with v2.5 paired-end, 150 bp chemistry. The processed negative controls did not yield any DNA and were not sequenced.

Seven genomic libraries (see Dataset S1 for the library types per sample) underwent an enrichment procedure by hybridisation capture using biotinylated probes supplied by MYcroarray (Ann Arbor, MI, USA; www.mycroarray.com). The capture utilised a subset of probes targeting 15,000 SNPs with the highest average per-sample coverage from the 1240k panel used in^56^. Prior to hybridisation, the DNA libraries (each ≈100 ng) were concentrated to dryness using a Speedvak concentrator (Savant) and resuspended in 6.8 µl of double-distilled water (ddH_2_O). Two rounds of enrichment were conducted according to the manufacturer’s protocol (v2.3.1) with minor changes^57^. Primers and IS5 and IS6 from^53^ as well as PISI and AIS4 from^57^, were used in the post-capture amplification of the libraries. The second pair was used to allow sequencing of one blunt-end Illumina library on an Ion Torrent Personal Genome Machine (Ion PGM) system (Ion Torrent, ThermoFisher Scientific Inc.). The library was then sequenced with the Ion PGM system at the Molecular Biology Techniques Laboratory, Faculty of Biology, Adam Mickiewicz University, using the Ion Torrent One Touch System II and the Ion One Touch 200 template kit v2 DL according to the manufacturer’s recommendations. Sequencing was performed on the Ion 318TM Chip Kit v2 using 520 flows and the Ion PGM Hi-Q sequencing kit v2.

### Processing of raw DNA sequence data and read mapping

The obtained paired-end Illumina sequence reads were first merged (with the minimum required overlap set to 11) and the adapters were removed using AdapterRemoval v2.1.7^58^ or MergeReadsFastQ_cc.py^59^. The reads acquired by Ion PGM sequencing were processed following a custom pipeline^57^.

Finally, trimmed and merged reads were mapped to the hs37d5 version of the human reference genome using Burrows-Wheeler Aligner v0.7.17-r1188^60^ with the following nondefault parameter settings: -l 16500 -n 0.01 -o 2^2,61^. Duplicate reads were detected and collapsed using a modified version of FilterUniqueSAMCons.py^59^. In addition, reads with reference identity <90% or read length shorter than 35 bp were removed.

### Basic statistics

The number of reads, the proportion of reads that mapped to the human genome, average read length, clonality, and mean depth of coverage were calculated to assess each library’s quality^52^. The ratio of reads that mapped to sex chromosomes was used to determine the genetic sex of each individual^62^. For samples that underwent multiple rounds of sequencing, the mapped bam files from each library were merged with the use of the merge option in samtools v1.5^63^. The merged bam files underwent duplicate read removal and basic statistic calculations were performed as in the case of single libraries.

### Contamination estimates

To assess the authenticity of the data, several methods were applied both for the individual and merged bam files. The signature of deamination at the 5′ and 3′ read ends^64^ and the read length distribution were calculated using MapDamage v2.0.8^65^.

ContamMix v1.0.10 ^66^ was used with default parameters to calculate the posterior probability of mtDNA contamination using a Bayesian approach. For this purpose, the reads were remapped against the rcrs mitochondrial sequence^67^, following the same procedure as in the case of the whole human genome, and consensus mtDNA sequences were called with the use of the *doFasta* tool of the ANGSD v0.910 package^68^; reads were accepted only if they had a mapping score of 30, a minimum base quality of 20, and positions with a minimum coverage of 3.

For samples determined to derive from males, X-chromosome-based contamination estimation was performed using the *contamination R* script included in ANGSD package. Any individual libraries and merged datasets for which contamination estimates exceeded 0.2 and/or the frequency of postmortem damage fell below 25% at the 5′- and 3′-ends were excluded from further analyses.

### Mitochondrial and Y-chromosome analyses

Consensus mtDNA sequences were called as described above for mtDNA contamination estimates. The obtained fasta files were used to assign mtDNA haplogroups utilising the Haplogrep^69^ online tool based on the PhyloTree phylogenetic tree build 17^70^.

For the individuals determined to be genetic males, the most likely Y-chromosome haplogroups (Y-DNA) were assigned based on the genotype calls from 69 391 no-indel branch determining SNPs obtained from the International Society of Genetic Genealogy collection (obtained in December 2021 from https://isogg.org/). The genotypes were called based on the genotype likelihood calculated with the use of the aDNA_GenoCaller.py script^71^, which takes into account the post-mortem damage estimated with MapDamage. The genotypes in low-quality (genetic quality [GQ] < 50) transition sites were used only when C or G alleles were found. When a T or A allele was found in transversion sites that did not have those alleles according to the reference dataset, they were assumed to be a result of post-mortem deamination and labelled C and G, respectively. In addition, where damage-repaired libraries were available, the transitions were also called with aDNA_GenoCaller.py script and merged with the genotype data.

Individuals were assigned to the lowest level haplogroup supported by the highest number of derived mutations linked to the most likely lineage starting from the Y-DNA tree root (Supplementary Data 4). The derived mutations not connected to this lineage were not taken into consideration.

### Datasets

Several modern datasets were used for various analyses:

Human Origins (HO):616 938 autosomal SNP sites from the Affymetrix Human Origins/The Human Origins SNP Array complete dataset containing genotypes for 2583 individuals representing 214 populations worldwide^72^ narrowed down to 1172 individuals representing 89 populations in Europe and northwestern Asia.

Simons Genome Diversity Project (SGDP): 1,001,613 autosomal nonfunctional SNPs with minor allele frequency (MAF) < 0.005 from the Simons Genome Diversity Project^73^ whole genome set matching 1,240,000 capture data available for a large portion of comparative ancient samples.

1k: 6,864,699 autosomal SNPs from the 1000 Genomes Project^74^

1k_trv_YRI: 1,622,524 autosomal transversions from the 1000 Genomes Project^74^, of which the minor allele frequency in the Yoruba (YRI) population was at least 0.1^55^.

1k_X:3,357,504 SNP from outside the pseudoautosomal region of the X chromosome from the 1000 Genomes Project^74^.

### Kinship analyses

Both READ^75^ and NgsRelate^76^ were used to estimate relatedness between pairs of individuals.

The genotype likelihoods for the SNPs from the 1k and 1k_X panels for NgsRelate analysis were calculated with the use of aDNA_GenoCaller.py for transversions in the merged bam files and aDNA_GenoCaller.py script for transitions if damage-repaired libraries were present^71^.

To maximise the number of overlapping SNPs while filtering them for linkage disequilibrium (LD), all possible combinations of individual pairs were merged and thinned separately with the vcftools^77^ (0.1.16)-thin 2500 option. Then, NgsRelate was used on each pair after annotating the vcf file with allele frequencies from the West Eurasia populations from the reference panel (Supplementary Data 18 and Supplementary Data 19).

The genotypes for the SNPs from the 1k panel for READ analysis were acquired from genotype likelihoods obtained as above and filtered similar to the Y chromosome genotypes. Then, READ was used on the haploidized final dataset after filtering out SNPs with a minimal allele frequency below 0.1 (Supplementary Data 20).

### Pedigree reconstruction

Only the kinship estimates based on at least 10,000 (or 1000, for the 1k_X panel) overlapping SNPs were considered. Age at death; genetic sex; R0, R1 and R2 values for autosomal data; the difference between θ values for autosomal and X-chromosome data; and mitochondrial and Y-DNA haplogroups were used to determine the exact kin relation where possible. In case of conflicting reconstructed degree of kinship between the two used methods the results of NGSrelate were found to be more reliable. The parent-offspring and sibling pairs were first distinguished with the uniparental markers and age at death (e.g. if both individuals were children or males sharing the same mitochondrial haplogroup, they were interpreted as siblings). Then, where available, the degrees of kinship to other related individuals were compared. For example, if an individual was found to share 1st-degree kinship with one individual from a pair of known siblings and 2nd-degree with the other, the individual in question was determined to be child of the first sibling and niece/nephew of the second. Similarly, if 2nd-degree kinship was detected with only one individual from a pair of known siblings, it was interpreted as a grandchild of this individual, as the grandparent, uncle or aunt, and niece or nephew would all be equally related to both siblings. Finally, to verify assumptions based on the abovementioned factors, or in the case of their absence, X chromosomal θ and autosomal k1 were used The summary of all the pairs of individuals found to be related can be found in Supplementary Data 21.

### Population genetics

#### Principal component analysis

The PCA was carried out as an initial assessment of the genetic affinities of the analysed populations. The *smartpca* program in the EIGENSOFT package^78^ was used to estimate eigenvectors in the HO dataset. The ancient samples that overlapped with at least 98% of the reference panel SNPs were projected onto the first two principal components inferred from modern samples with the following nondefault settings: altnormstyle: NO, numoutlieriter: 0, numoutlierevec: 0, lsqproject: YES, shrinkmode: YES.

To assess, whatever the use of capture data in the five cases where it was merged with shotgun data could cause any bias, in three cases where sufficient amount of both types of data were present, they were analysed both separately and combined (see results in Supplementary Information text).

### Admixture analysis

Unsupervised model-based clustering was performed with the use of ADMIXTURE (v1.3) software^79^ on the same set of individuals as included in the PCA. Prior to the analysis, the dataset was first limited to transversions and then pruned for LD with the use of the–indep-pairwise 200 25 0.4 option from PLINK toolset (v1.90b5)^80^. Finally, clustering was performed on samples that overlapped with at least 85% of the final set of 70 149 SNPs for K = 3 to K = 14 in 10 replicate runs with different random seeds for each K. The results were visualised with the use of pong v1.4.9^81^ to bundle together the membership coefficient matrices (Q) from different replicates and the different numbers of clusters. The K values with the smallest standard error of the cross-validation error estimate for the selected set of individuals (K = 7, CV = 0.559698) were then selected and discussed in detail; the results for all K values of all samples can be found in the Supplementary Materials (Supplementary Data 11 and Supplementary Fig. 6).

### F3 and D statistics

Various analyses were performed to quantify shared drift between individuals and/or populations as their divergence from an outgroup population with the use of outgroup *f3-* and *D*-statistics, implemented with the *qp3pop* and *qpDstat* tools from ADMIXTOOLS^82^ software, respectively. All analyses were performed on data genotyped to the 1k_trv_YRI reference dataset, using the African Yoruba population as an outgroup.

Individuals determined to be genetic outliers, based on their position on PCA space and admixture analysis results, were not included in the determination of populations based on individuals’ association with archaeological cultures or horizons. In addition, only one individual (with the highest genome coverage) was selected from each group of individuals determined to share direct genetic kinship.

Before running the analyses, variants found in only one ancient individual and individuals with <85% genotyped SNPs were filtered out. First, *qp3pop* with default settings was used to test individuals’ assignment to populations in the form of *f3*(YRI, ancient population, ancient population) and *f3*(YRI, tested individual, ancient population). Then, shared drift between pairs of individuals, except for 1st- and 2nd-degree relatives, was measured in each population to assess within-group genetic diversity^83^ in the form of *f3*(YRI, tested individual, tested individual). The results (Supplementary Data 9) were displayed as boxplots of 1-*f3* values for all individual representative populations analysed here and several Bronze Age reference populations (Fig. 2C). To determine how data from TC sites with a high number of directly related individuals affected the obtained results, the sites yielding the most individuals (Żerniki Górne, Pielgrzymowice and Gustorzyn) were plotted separately from the rest of the TC individuals.

To determine if sex bias was present in the gene flow from the putative source of hunter-gatherer ancestry during the formation of the TC, the data was also genotyped to the 1k_X dataset, which represented the X chromosome of the 1 000 Genome Project and was filtered similar to the autosomal data. Then, *qp3pop* was run on both autosomal and X-chromosome data in the form of *f3*(YRI, TC individual, WHG) to track the temporal changes, and *f3*(YRI, TC, pop X) for direct comparison on population level (where various populations with dominating Hunter-Gatherer, steppe or farmer ancestry were used as pop X)^15^. The temporal changes were displayed on graphs for which trend lines were obtained with geom smooth tool from ggplot2 package in R. The conditional means were calculated both for all the individuals and after removing outliers and displayed with 95% confidence intervals.

*qpDstat* with default settings was applied to verify various demographic scenarios by testing the analysed individuals against pairs of potential ancestry sources in the form of *D*(YRI, tested individual, population 1, population 2). This analysis was used to determine which ancient population out of all the analysed populations was the best proxy for the source of hunter-gather ancestry in the TC and post-CWC populations (Supplementary Data 12). In addition, to confirm the predominance of patrilocality in TC groups, data from individuals from the Żerniki site were tested in the form of *D*(YRI, Żerniki individual, Żerniki population, other TC individuals), and the results were plotted separately for each sex (Supplementary Data 10, Fig. 2D).

### qpAdmixture

Based on the *f3* and *D* statistic results, various demographic scenarios of TC origin were tested with the use of *qpAdm* tool from the ADMIXTOOLS^82^ package with the following nondefault parameters: details: YES, summary: YES, allsnps: YES, and maxrank: 7. The SGDP reference dataset was used, and additional ancient individuals were included as outgroups. The right file with the outgroup population comprised the Onge, Papuan, Han, Mixe, Karitiana, Natufian, and Chukchi populations from the dataset and 25 ancient individuals from 13 hunter-gatherer populations worldwide with addition of Neolithic Anatolians (Supplementary Data 3). Two- and three-way admixture models were tested. The models on population levels included the TC and KC as test populations. The two-way models additionally included one Early Bronze Age post-CWC and one hunter-gather population; the three-way models also included a late Neolithic population from Eastern Europe, characterised by a mixture of Anatolian farmer and hunter-gatherer ancestry. All results are presented in Supplementary Data 13 and Supplementary Data 14; only cases where the full model could be separated from the nested models were considered and discussed further. Based on the results 16 populations were chosen to tun the analysis in more discriminative rotation outgroup approach as suggested by^37^. The models in which the source populations were comprised of only single individuals or had a nested *p* value > 0.05 were reported however approached with caution and not considered plausible (Supplementary Data 15 and Supplementary Data 16). In addition, individual levels of admixture were calculated with the use the three-way models (Supplementary Data 17). The degree of WHG ancestry for individuals published here and individuals from selected Neolithic and Bronze-Age reference populations was calculated using a three-way model including the WHG, Anatolian Neolithic (AN) and Yamnaya Culture (YAM) populations as source populations. In cases where the model had a nested *p* value > 0.05. A separate analysis was performed for the corresponding two-way model. When the nested model did not include WHG, the ancestry was set to 0 (Supplementary Data 17). To track the temporal changes in the amount of hunter-gatherer ancestry the coefficients values for WHG, for which radiocarbon dates were available, were plotted in R together with conditional means similarly to the *f3* values.

### Reporting summary

Further information on research design is available in the Nature Portfolio Reporting Summary linked to this article.

## Supplementary information

Supplementary Information

Description of Additional Supplementary Files

Supplementary Datasets 1-21

Reporting Summary

## Data Availability

The aligned sequencing data have been deposited in the European Nucleotide Archive database under accession number PRJEB53670.
