# Supplementary material for: Patrilocality and hunter-gatherer-related ancestry of populations in East-Central Europe during the Middle Bronze Age
Source: Nat Commun. 2023 Aug 1;14:4395. doi: 10.1038/s41467-023-40072-9 (PMC10393988; doi:10.1038/s41467-023-40072-9)
Supplement: Supplementary file 1 — Supplementary Information [file 41467_2023_40072_MOESM1_ESM.pdf]

Supplementary Information

Patrilocality and hunter-gatherer-related ancestry of populations in East-Central Europe during the Middle Bronze Age

Maciej Chyleński[1]*, Przemysław Makarowicz[2], Anna Juras[1]*, Maja Krzewińska[3,4], Łukasz Pospieszny[5,6], Edvard Ehler[7], Agnieszka Breszka[1], Jacek Górski[8,9], Halina Taras[10], Anita Szczepanek[11], Marta Polańska[12], Piotr Włodarczak[11], Anna Lasota-Kuś[11], Irena Wójcik[9], Jan Romaniszyn[2], Marzena Szmyt[2,13], Aleksander Kośko[2], Marcin Ignaczak[2], Sylwester Sadowski[10], Andrzej Matoga[9], Anna Grossman[14], Vasyl Ilchyshyn[15], Maryna O. Yahodinska[16], Adriana Romańska[17], Krzysztof Tunia[11], Marcin Przybyła[18], Ryszard Grygiel[19], Krzysztof Szostek[20], Miroslawa Dabert[21], Anders Götherström[3,4], Mattias Jakobsson[22,23,24] & Helena Malmström[22,23]*

1. Institute of Human Biology and Evolution, Faculty of Biology, Adam Mickiewicz University in Poznań, Uniwersytetu Poznańskiego 6, 61-614 Poznań, Poland

2. Faculty of Archaeology, Adam Mickiewicz University in Poznań, Uniwersytetu Poznańskiego 7, 61- 614 Poznań, Poland

3. Archaeological Research Laboratory, Department of Archaeology and Classical Studies, Stockholm University, Lilla Frescativägen 7, SE-106 91 Stockholm, Sweden

4. Centre for Palaeogentics, Svante Arrhenius väg 20C, SE-106 91 Stockholm, Sweden

5. Institute of Archaeology, University of Gdańsk, ul. Bielańska 5, 80-851 Gdańsk,and Ethnology, Polish Academy of Sciences, Rubież 46, 61-612 Poznań, Poland

6. Department of Anthropology and Archaeology, University of Bristol, 43 Woodland Road, Bristol BS8 1UU, United Kingdom

7. Laboratory of Genomics and Bioinformatics, Institute of Molecular Genetics of the Czech Academy of SciencesASCR, v. v. i., Vídeňská 1083, 142 20 Prague 4, Czech Republic

8. Department of History and Cultural Heritage, University of Pope Jan Paweł II, Kanonicza 9, 31-002 Cracow, Poland.

9. Archaeological Museum in Cracow, Senacka 3, 31-002, Cracow, Poland

10. Institute of Archaeology, Maria Curie-Skłodowska University, M.C.-Skłodowska sq. 4, 20-031 Lublin, Poland

11. Institute of Archaeology and Ethnology, Polish Academy of Science, Sławkowska 17, 31-016 Cracow, Poland

12. Department of Material and Spiritual Culture, Lublin Museum, Zamkowa 9, 20-117 Lublin, Poland

13. Archaeological Museum in Poznań, Wodna 27, 61-781 Poznań, Poland

14. Muzeum Archeologiczne w Biskupinie, Biskupin 17, 88-410 Gąsawa, Poland

15. Zaliztsi Museum of Local LoreZaloztsy Muzey, Schevchenka 51, Zalizhtsyi, Ternopil oblreg. 47243, Ukraine

16. Ternopil Regional Center for Protection and Research of Cultural Heritage Sites, Kyyivs′ka 3a, 46016 Ternopil, Ukraine

17. Wojewódzki Urząd Ochrony Zabytków, Gołębia 2, 61-840 Poznań, Poland

18. Archaeological company "Dolmen Marcin Przybyła, Michał Podsiadło s.c.", Serkowskiego Sq. 8/3, 30-512 Cracow, Poland

19. Museum of Archaeology and Ethnography in Łódź, Plac Wolności 14, 91-415 Łódź

20. Institute of Biological Sciences, Cardinal Stefan Wyszynski University in Warsaw, Wóycickiego 1/3, 01-938 Warsaw, Poland

21. Molecular Biology Techniques Laboratory, Faculty of Biology, Adam Mickiewicz University in Poznań, Uniwersytetu Poznańskiego 6, 61-614 Poznań, Poland

22. Human Evolution, Department of Organismal Biology and SciLifeLab, Uppsala University, Norbyvägen 18C, SE-752 36 Uppsala, Sweden

23. Centre for Anthropological Research, University of Johannesburg, Auckland Park, 2006 Johannesburg South Africa

24. SciLifeLab, Stockholm and Uppsala, Sweden

*Corresponding authors: Maciej Chyleński, PhD, tel.: +48 61 829 56 25, e-mail: maciej.ch@amu.edu.pl; Anna Juras, PhD, tel.: +48 61 829 56 25, e-mail: annaj@amu.edu.pl; Helena Malmström, e-mail: helena.malmstrom@ebc.uu.se

Table of content

Materials and archaeological background 4

The Iwno culture 4

The Mierzanowice culture 7

The Strzyżów culture 13

The Trzciniec culture 17

The Komarów culture 33

Influence of the 15k capture on the data analysis 36

Supplementary Figures 38

Supplementary References 43

Materials and archaeological background

The Iwno culture

At the Polish Lowlands, in the mid of 3rd millennium BCE, the Iwno culture (IC) emerged, a syncretic unit that combines traits of Bell Beakers, north European variants of Corded Ware Culture and, to a lesser degree, early Únětice culture [1–4]. It belongs to the Northern European Province of the Bell Beakers and is dated, on the basis of radiocarbon (^{14}C) dates, between 2500/2400 and 1800 BCE (Early Bronze Age [5,6]).

The IC is known from over 350 sites situated in the regions of Kujawy, Pałuki, Eastern Pomerania, Chełmno Land and Great Poland [6]. The oldest IC shows syncretism of the Bell Beakers and the late Single Grave culture. Mainly in its first two phases, between the middle and the end of the 3rd millennium BCE, it shows the presence of 'true' Bell Beaker traits. In the third phase, between 2050/2000 and 1800 BCE, the IC underwent a certain "únětization" process observable, for instance, in the adaptation of Únětice culture-style metal items buried as grave goods, and in the occurrence of one-type and multi-type hoards [3,7].

Most likely, the IC settlements occupied an area of 800-2000 m^2. Typical of such settlements are light, seasonal post-made shelters and sunken-floor houses. Single-house hamlets occurred very often, their size suggests that they were inhabited by nuclear families. It cannot be ruled out that larger communities consisted of 3-4 nuclear families or 1-2 extended families composed of three generations. Most likely, the settlements existed for a short period of time: they were single- or several-season structures. Only in a few cases one can talk about long-term multi-phase occupation of a settlement. The IC-associated communities were relatively mobile. The basis of their economy was animal raising, especially cattle-breeding [3,6].

Settlements are frequently accompanied by cemeteries. The IC funerary ritual generally reflects the patterns of the Bell Beakers, though they differ from one another in some details. However, it must be taken into account that the available data comes from few graves with anthropologically assessed human skeletal remains. Small cemeteries, comprising several up to over a dozen flat graves are typical. In the early phases, stone-packing graves and stone-timber structures are found. The dead were buried in the same manner for nearly 700 years: in oval pits, uncremated, usually singly in a crouched position with legs strongly contracted and hands bent at elbows. Graves containing several individuals are found as well, with skeletons

occasionally being incomplete. In the existing cases no major differences can be observed in furnishing with grave goods that should point to a particular treatment of one of the sexes [6]. Altogether, we sampled bones from 7 individuals for DNA extraction. Eventually, we obtained nuclear genomes from 3 individuals that are described below.

Cemeteries, graves, and individuals of the Iwno culture

Gąsawa, site 6, Żnin district, Kujawy-Pomorze province, Poland

The Early Bronze Age grave complex was located on the eastern slope of Lake Gąsawa valley in the Pałuki region [8]. It comprised three burials (graves 5, 6 & 7) furnished with two bowls and three beakers. A complete skeleton has found only in grave 5.

poz462: Feature 5. The burial pit measured 2.3 × 2 m), remains of an adult male (40- 50-year-old) were discovered at its eastern wall. The individual lied in a contracted position on the right side along the N-S axis, with head pointing S and facing E. Two small stones were found on his both sides. The third one, slightly larger, was placed below the lower limbs. A beaker and animal bone (cattle) were found below the feet. Human bone sample was AMS radiocarbon dated to 2008-1750 BCE (Poz- 86828, 3540±35 BP). A tooth was sampled for DNA analyses. We show that this was a male, carrying mtDNA haplogroup U4b1a1a and Y-chromosomal haplogroup R1b1a1b.

Łojewo, site 4, Inowrocław district, Kujawy-Pomorze province, Poland

A multicultural site, located in at the edge of the Szarlej Lake valley in the Kujawy region [9].

poz502: Feature 37. The burial pit, oriented along NW-SE axis, had an irregular oval shape and dimensions of 1.65 × 0.95 m. It contained remains of three individuals: an adult male (30-35-year-old) (poz502) and two females, both of 20-25-year-old [9]. Disarticulated bones of both females were placed in the northern part of the grave. The male lied in a contracted position on the right side. Skeletal remains of animals (cattle, sheep/goats and pigs) were found among human bones. In the northern part of the burial pit, two vessels (a vase and a pot) and stone grinder were found. The male was AMS radiocarbon dated to 2008-1750 BCE (Poz-40392, 3540±35 BP). The petrous part of a temporal bone was sampled for DNA extraction. The results of this study show that this was a male who belonged to mtDNA haplogroup K1b1b1 and Y-

chromosomal haplogroup haplogroup T1a1. This haplogroup was previously found only in Chalcolithic samples from Levant [10] and Neolithic samples in Bulgaria [11]. This further supports the interpretation of this individual as originating in isolated population of Neolithic descent.

Siniarzewo, site 1, Aleksandrów Kujawski district, Kujawy-Pomorze province, Poland

A multicultural site located on the Inowrocław Plain in the Kujawy region [2,12].

poz929: Feature H21. The grave was disturbed by a later trench [12]. It contained a skeleton of 12-15-year-old female, laying in the contracted position on the left side, along N-S axis, with head pointing N and facing SE [13]. The burial was equipped with two bowls. This individual was AMS radiocarbon dated to 1941-1746 BCE (Poz-92318, 3520 ± 35 BP) [14]. The petrous part of temporal bone was collected for DNA extraction. We show that this was a female, carrying mtDNA haplogroup N1a1a1a2.

The Mierzanowice culture

The Early Bronze Age Mierzanowice culture (MC) developed between 2400/2350 and 1600 BCE [15–17]. It covered the uplands of modern-day south-eastern Poland, adjacent regions of south-western and eastern Slovakia and Ukraine (Volhyn-Podole Upland), Upper Silesia and Moravia. The MC-associated communities settled mostly in loess uplands. Beginning with the late 3rd millennium BCE, they formed micro-regional settlement structures, covering from a few up to 20 km^2. Stable settlement were hubs in the centre of those micro-regions. Usually, settlements existed over several phases and in some cases were inhabited for several hundred years. In their vicinity, less stable settlements and cemeteries appeared. Permanent settlements were located on edges and slopes of river valleys, frequently in naturally defensible locations. The MC-associated communities were engaged in mixed farming [15,18].

Cemeteries were located close to large settlements and had from several dozen to several hundred flat graves arranged in rows [19]. Graves were occasionally dug into older Corded Ware culture burial mounds or placed on their circumference [20]. The dead were usually individually buried in rectangular or oval pits. Males were laid on their right side, with the head pointing W or S, while females on their left, with the head pointing E or N [21]. There are also few instances of double burials, in most cases of females with children; only exceptionally collective burials were found. Some graves contained traces of wooden coffins or other organic containers [22,23]. Grave goods are thought to be sex-dependent and included pottery vessels, flint and stone tools, ornaments and dress accessories made of bone, shell, faience and copper. Rich burials furnished with prestige objects (e.g. faience-bead necklaces or copper willow-leaf ear wraps) were found but also pottery-free and unfurnished graves are known [19,23–25]. Occasionally traces of manipulation of human remains were observed such as decapitation, severance of limbs, and partial or secondary burials [23,25].

The social structure of the MC populations is thought to be centred around the 'male/female' opposition, while its hierarchic structure can be best described as moderate [16]. Bronze, practically, was not used in spite of the fact that MC-associated communities were located in close vicinity to large bronze-production centres in the Carpathian Basin and in the impact range of the Únětice culture.

Altogether, we sampled bones from 25 MC individuals for DNA extraction. In the end, we obtained nuclear genomes from 15 individuals that are described below.

Cemeteries, graves and individuals of the Mierzanowice culture

Hrebenne, site 10, Hrubieszów district, Lublin province

The site was located on the edge of Horodło Ridge in Volhyn Upland, at a small nameless watercourse. Thirteen features were unearthe, includong: two pits and a grave of the MC, and three Strzyżów culture graves.

poz790 and poz791: Grave 3/97 is a well-preserved feature discovered approx. 0.25 m below the present ground level. In plan, it was roughly circular, 2.15 × 1.95 m in size. Slightly above the bottom of the pit a collective burial was found. The centre and eastern part of the pit contained skeletons of three children (A-C), while at the north wall a cluster of human skulls fragments and an animal skeleton were found. The skeletons of older children (individuals A and B or poz790 and poz791, respectively) lay parallel to each other along the W-E axis with the heads pointing W. The skeleton of the third and the youngest child (individual C) laid along the NW-SE axis with its lower limbs contracted underneath the torso of individual B. The skeletons were incomplete with some bones displaced. Animal bones were found next to individuals A and B [26]. Both individuals were previously AMS radiocarbon dated to 2290-2038 BCE (poz790; Poz-101471, 3760 ± 35 BP) and 2282-2028 BCE (poz791; Poz-101473, 3735 ± 35 BP) [27]. The petrous parts of temporal bones were sampled for DNA extraction. As in the previous study [27], we confirm that both individuals belonged to the mtDNA haplogroup U5a1i. In this study, we show that individuals poz790 and poz791 were a male and a female, respectively. Poz790 was assigned to Y-chromosomal haplogroup R1a1a1~. The two individuals were found to share 2nd degree kinship. Based on the fact both individuals were children and shared mtDNA haplogroup the pair was identified to be either a uncle/niece or aunt/nephew.

Mokrzec, no site number, Przysucha district, Maszowsze province, Poland

The site was located on South Mazovian Hills, at the border between Masovian Lowland and Lesser Poland Upland, on a hill rising above a valley of an unnamed stream, a tributary of the Wiązownica River. Human skeletal remains were accidentally discovered in 1994 [28].

poz1039: Single grave contained human remains of a ~30-years-old male (MOR/A/1444), probably on the right side. Grave goods included a small cup, items made of copper (a bracelet, five chapes, blade of a dagger), a bone awl, and flint tools and flakes [28]. This individual was

AMS radiocarbon dated to 2206-2134 BCE (Poz-31598, 3755 ± 55 BP) [28]. The petrous part of a temporal bone was collected for DNA extraction. In this study we show that individual poz1039 was a male who belonged to mtDNA haplogroup H3b+16129 and Y-chromosomal haplogroup R1a1a1~.

Pieczeniegi (*de facto* Rzemiędzice), no site number, Miechów district, Małopolska province

This site is located in the Miechów Upland, part of the Lesser Poland Upland, on a terrace of the Nidzica River. It is a multicultural settlement with MC and Lusatian culture cemeteries [29]. Rescue excavations preceding a house construction took place in 1960. As a result, six MC graves were found. The name Pieczeniegi refers to a part of the village of Rzemiędzice.

poz538: The oval-like burial pit 2 measured 2.30 × 0.80 m and extended along the W-E axis. On its bottom, a crouched skeleton lying on its left side, with the head pointing E was found. The left hip was girded by a string of cylindrical bone beads [29]. This individual was AMS radiocarbon dated to 2031-1781 BCE (Poz-104946, 3580 ± 35 BP) [27]. The petrous part of bone was sampled for DNA extraction. As in previous study [27], we show that this individual carried mtDNA haplogroup T2e1. In this study we assigned poz538 as female.

Świniary Stare, site 1, Sandomierz district, Świętokrzyskie province, Poland

This is a multicultural site located in the Sandomierz Upland, part of Lesser Poland Upland, on a sandy terrace of the old river bed of the Vistula, it was rescue excavated in 1959-1962 prior to opening a sand quarry [30]. Fifty-two inhumations were identified and dated to the classic phase of the MC.

poz514: Grave 7 contained fragments of skeleton belonging to an adult male, lying along the W-E axis, with the head pointing W. The legs were flexed and the trunk was in a supine position, turned slightly to the right side. The deceased was furnished with a single tool made from a chocolate-flint flake [30]. Basing on typochronology the grave was dated to 2050-1850 BCE. The petrous part of temporal bone was sampled for DNA analyses. As in the previous cases [27], we confirm that this individual belonged to mtDNA haplogroup X2b4. In this study we assigned poz514 to Y-chromosomal haplogroup R1a1a1b~.

poz529 and poz530: A badly preserved grave 44 with remains of two individuals. Judging by their position, the deceased must have lain on the right side, with the head pointing E [30]. Individual poz529 was AMS radiocarbon dated to 2030-1776 BCE (Poz-104950, 3575 ± 35) [27], while poz530 was dated to 2050-1850 BCE basing on typochronology. The petrous parts of temporal bones were collected from both individuals for DNA extraction. As in our previous analyses [27], poz529 and poz530 were found to belong to mtDNA haplogroups I1a1a and K2a, respectively. In this study we show that both individuals were females.

poz531: Grave 50. A disturbed burial in which only a damaged skull of an adult female was found. Judging by its position, the deceased must have lain on the left side, with the head pointing E [30]. This individual was AMS radiocarbon dated to 2127-1883 BCE (Poz-104952, 3610 ± 35 BP) [27]. The petrous part of a temporal bone was sampled for DNA analyses. As in our previous analyses [27], we confirm that poz531 belonged to mtDNA haplogroup I1a1a. In this study we assigned this individual as a female. The individual was found to be either the same individual or identical twin of poz529. As the sampled temporal bones were different (left and right respectively) and the remains were stored in different storage boxes neither scenario can be confirmed.

poz533: Grave 53 contained remains of an adult male, lying on the right side, along the W-E axis, with the head pointing W. The legs were slightly flexed and one arm slightly protruded forward. The grave contained only a flint flake [30]. This individual was AMS radiocarbon dated for this study to 2132-1900 BCE (Poz-142774, 3635 ± 30 BP). We assigned the same mtDNA haplogroup for poz533 (U5b2a2c) that confirmed our previous analyses [27].

poz534: Grave 55 contained a fragmentary preserved skeleton of adult male. It was situated on the W-E axis, with head pointing W and facing S [30]. There were no grave goods in the burial pit. Basing on typochronology this individual was dated to 2050-1850 BCE. The petrous part of a temporal bone was collected for DNA extraction. Our genetic results show that this was a male who belonged to mtDNA haplogroup H5a1 and Y-chromosomal haplogroup R1a1a1~.

poz535: Grave 56. A heavily disrupted burial in which only a skull of an adult male was preserved. Judging by its position, the skeleton must have lain on the right side, with the head pointing W [30]. Basing on typochronology this grave was dated to 2050-1850 BCE. The petrous part of a temporal bone was collected for DNA analyses. As in previous study [27], individual

poz535 was found to belong to mtDNA haplogroup U5a2c3. In this study we show that this individual was a female.

poz537: A partially disrupted grave 59 contained the skeleton of an adult male, lying on the back and slightly turned to the right, along the W-E axis, with the head pointing. The legs were drawn up while the arms were placed to the sides. A chocolate-flint arrowhead, bone spacer and a damaged wild-boar tusk were found in the grave [30]. This individual was AMS radiocarbon dated to 2128-1886 BCE (Poz-83841, 3615 ± 35 BP) [27]. The petrous part of a temporal bone was collected for DNA extraction. As in previous study [27], individual poz537 was determined to belong to mtDNA haplogroup W6a. Our genetic results show that this was a male who belonged to Y-chromosomal haplogroup R.

Zubowice, site 1A, Zamość district, Lublin province, Poland.

The is a multicultural site located in in the Hrubieszów Basin, part of Volhynia Upland, on a sandy elevation in the valley of Sieniocha River, a left tributary of Huczwa. Single (grave 1) and collective MC inhumation graves were found at this site (Sadowski 2006; Kłosińska et al. 2010). The latter comprised five individuals placed in a circle in vast pit 14 (feature 15/grave 3, feature 16/grave 4, feature 21/grave 5, feature 22/grave 6, feature 25/grave 7). The better preserved remains suggest that the deceased laid in a crouched on the side.

poz557: Pit 14, feature 15, grave 3. It contained the skeleton of an adult male (*Adultus/Maturus*), in a contracted position, on the right side, along the E-W axis, with the head pointing W. The skull bored a green trace left by a decomposed copper ornament. No other grave goods were found [31]. This individual was AMS radiocarbon dated to 2193-1950 BCE (Poz-104948, 3675 ± 35 BP [27]. The petrous part of a temporal bone was collected for DNA extraction. As in previous study [27], individual poz557 was found to belong to mtDNA haplogroup I1a. In this study we show that this was a male who belonged to Y-chromosomal haplogroup I2a1b1.

poz559: Pit 14, feature 22, grave 6. It contained the skeleton of a female (35-45-year-old) in a highly contracted position, on the left side, with head pointing N. The hands were strongly bent at the elbows and placed under the face. At the skull, a scraper-knife made of Volhynian flint was found [31]. Basing on typochronology this individual was dated to 2050-1850 BCE. The petrous part of a temporal bone was sampled for DNA analyses. Our genetic results show that

this was a female who belonged to mtDNA haplogroup H13a1a2. The individual was found to share first degree kinship with poz556, although the estimation is based on low number of SNPs and is therefore not completely reliable.

poz556: Pit 14, feature 25, grave 7, contained the skeleton of an adult male (*Maturus*), on the right side, along the N-S axis. Grave goods included burned and broken stone shaft-hole axe (placed by the skull). The skull bored a green trace left by a decomposed copper ornament [31]. Individual poz556 was AMS radiocarbon dated to 2340-2042 BCE (Poz-104947, 3780 ± 35 BP) [27]. The petrous part of a temporal bone was sampled for DNA extraction. As in previous study [27], we found that individual poz556 belong to mtDNA haplogroup H13a1a2. In this study we show that this was a male who belonged to Y-chromosomal haplogroup R1a1a1b1. The individual was found to share first degree kinship with poz559, although the estimation is based on low number of SNPs and is therefore not completely reliable.

The Strzyżów culture

The Strzyżów culture (SC) developed in the Early and Middle Bronze Age, between 2000 BCE and 1600 BCE. It covered the present-day Polish-Ukrainian borderland: between the Wieprz River (right tributary of the Vistula) in the west and the Horyn River (right tributary of the Pripet) in the east [16,19]. This area included the northern part of the Volhyn Upland (Horodło Ridge and Hrubieszów Basin) and a part of the Lublin Upland and Volhyn Polesie [32,33]. Recently, a suggestion has been made to extend its range north (to Western Polesie) and south (to Roztocze Upland) [19]. SC-associated communities settled on loess uplands, settlements were located on high headlands and valley ridges. Their subsistence was based on mixed farming which included crop cultivation and open-range animal raising [32,33].

Cemeteries were located in prominent places, close to settlements, and included from several to up to 20-30 inhumation graves [33–36]. Mounds were built over graves or they were dug into older barrows. The dead were laid in rectangular or oval pits, in an extended or slightly flexed position, usually along the E-W axis, with the face turned S [19,35,36]. Partially burned skeletons [34] and occasionally remains of wooden structures were found in some burials [37]. In the borderland between the SC and MC, exceptions to this rule were noticed. Single burials dominate, but double ones are known as well. Stone structures were encountered in a few cases, including pavements and kerbs, while timber structures are exceedingly rare [19].

Both richly furnished burials, containing prestige objects, and unfurnished ones were discovered. Grave goods, usually rich and diverse, include pottery vessels, flint and stone tools, and ornaments and dress accessories mostly made of bones, shells, faience and copper. Traces of mutilation of human remains were observed, e.g. decapitation and limb severance [36].

Altogether, we sampled bones from 27 individuals for DNA extraction. In the end, we obtained nuclear genomes from six individuals that are described below.

Cemeteries, graves, and individuals of the Strzyżów culture

Hrebenne, site 31, Hrubieszów district, Lublin province

The site is located within Horodło Ridge, part of Volhyn Upland, and occupied the edge of a headland in the valley of a nameless watercourse. Among 29 discovered features, there were 28 graves (SC, MC and undated ones) and one partially recognized settlement pit. At least two of the graves can be associated with SC [38].

poz788: Grave 3/98. The irregular burial pit measured 3.96 × 2.64 m and was oriented along W-E axis. It contained a skeleton determined to belong to a 20-30-year-old female (*Adultus*) [39] in an extended and supine position along the W-E axis with the head pointing W. At the bottom of the burial pit remains of a wooden structure were found. The grave goods included five pottery vessels, 10 flint artefacts (including an arrowhead and a retouched blade), copper wire ornament, 12 faience beads and five pendants made of animal teeth, and fragments of boar tusk. In addition, seven flint scarps were found on the bottom of the burial pit. This individual was AMS radiocarbon dated to 1941-1746 BCE (Poz-104938, 3520 ± 35 BP) [27]. The petrous part of a temporal bone was sampled for DNA extraction. As in previous study [27], individual poz788 was determined to belong to mtDNA haplogroup U5a1a2a. In this study we show that, in contrast to the anthropological interpretation, this was a male assigned to Y-chromosomal haplogroup R1b1a1b1a(1a2c1a1a1a1a1~).

Hrebenne, site 34, Hrubieszów district, Lublin province, Poland

A multicultural site located in Grzęda Horodelska, part of Volhyn Upland, on the slope of a prominent headland rising above the bottom of the valley of the nameless watercourse. Eight features were unearthed here, including two graves of the SC.

poz792: Grave 1/97. It contained an incomplete skeleton of a 25-30-year-old male. The deceased was placed in a supine position on the back with the head pointing W. 14 items were found in the burial pit, including elements of (probably incomplete) grave goods: a pottery vessel, three flint arrowheads, a copper bracelet, a shell pendant, fragments of two plates and three pendants made of a boar tusk, and three faience beads [38]. This individual was AMS radiocarbon dated for this study to 1882-1687 BCE (Poz-142762, 3455 ± 30 BP). The petrous part of a temporal bone was collected for DNA extraction. In this study we show that this was a male, carrying mtDNA haplogroup T2a1a and Y-chromosomal haplogroup R1a1a~.

Nieledew, site 1, Hrubieszów district, Lublin province, Poland

A multicultural sitelocated in the Hrubieszów Basin, part of Volhyn Upland, on the northern slope of a gentle loess elevation, slightly below its summit. In six SC graves, few poorly preserved human skeletal remains were found [40].

poz787: Grave 5 was badly damaged making the outline of the burial pit indiscernible. It contained a skeleton of an individual aged *Iuvenis*, lying with the head pointing NW. The grave goods comprised faience beads (53) and copper objects (two?) that left green traces on the zygomatic bone and a vertebra fragment [40]. This individual was AMS radiocarbon dated to 1935-1742 BCE (Poz-107535, 3510 ± 35 BP) [27]. The petrous part of a temporal bone was sampled for DNA extraction. We show, as in previous study [27], that individual poz787 belong to mtDNA haplogroup H6a1a. Our genetic results show that this was a female.

Raciborowice Kolonia, site 2, Chełm district, Lublin province, Poland

A multicultural site located at the edge of the Dubienka Depression, part of Volhyn Polesie, on a small headland protruding into the narrow valley of a nameless watercourse [34,37]. It included the largest SC cemetery known by now with 27 graves and relics of the above-ground building probably connected functionally with them.

poz794: Grave 11. A rectangular burial pit 0.80 × 1.95 m in size oriented along W-E axis. It contained the skeleton of 14-year-old individual (*Infans II*), in an extended prone position, along W-E axis with the head pointing W, equipped with a pottery vessel [34]. This individual was AMS radiocarbon dated to 1921-1697 BCE (Poz-142720, 3495 ± 35 BP) [27]. The petrous part of a temporal bone was collected for DNA extraction. As in previous study [27], individual poz794 was found to belong to mtDNA haplogroup T2a1b1a2. In this study we show that this was a male assigned to Y-chromosomal haplogroup R1a1a1b1a2b3a(1c2b~).

Stryjów, site 30, Krasnystaw district, Lublin province, Poland

The site is located in Działy Grabowieckie, the highest part of the Lublin Upland, on the summit of a loess headland, jutting into the valley of the Wolica River, a right-bank tributary of Wieprz. Two Early Bronze Age barrows were discovered here. In barrow 1 and in its eastern surroundings, nine graves were found [41].

poz230: Grave 8 was located east of barrow 1, in a depression covered with erosion strata. It had a stone construction, 2.45 × 0.80 m in size, and oriented along the N-S axis. On its bottom, lose human teeth and long bones were found, belonging to an individual aged *Adultus* (20-25 years), most likely a male. The deceased laid in a supine position on the back with the head pointing N, and legs flexed and slightly turned to the right. The grave inventory included a

copper ear band, two flint arrowheads, boar tusk fragments, two faience beads and 34 annular beads made of mollusc shells [41]. This individual was AMS radiocarbon dated to 2010-1769 BCE (Poz-66520 3545±30 BP) [41]. The petrous part of a temporal bone was sampled for DNA extraction. As in previous study [27], individual poz230 was found to belong to mtDNA haplogroup HV6. In this study we show that this was a male carrying Y-chromosomal haplogroup R1a1a1~.

Strzyżów, site 27, Hrubieszów district, Lublin province, Poland

A heavily damaged SC cemetery (human bones and other artefacts visible on the surface of the site and in the topsoil) located on Horodło Ridge, part of Volhyn Upland, on the edge of the Bug River valley [42].

poz758: Grave 1. No outline of the burial pit was observed. It contained a skeleton of male (*Maturus*), lying in a supine position on its back, along SE-NW axis with head pointing SE. Grave goods included fragments of three heavily damaged pottery vessels found to the left from the skull [43]. Basing on typochronology this grave was dated to 2000-1800 BCE. The petrous part of a temporal bone was collected for DNA extraction. In this study we show that this was a male assigned to mtDNA haplogroup I2 and Y-chromosomal haplogroup I(2a1a1b~).

The Trzciniec culture

The Trzciniec culture (TC) or the Trzciniec cultural circle (TCC) was one of the most extensive archaeological units of the Bronze Age. It existed between 1800 and 1100 BCE at the borderland between Central and Eastern Europe, mainly in the zones of primeval forest and forest steppe [44]. The TC-associated societies spread between Prosna and Warta River Basins in the West and Desna and Seym Rivers in the East, the lower Vistula and Nemen in the North and the middle Dniester in the South [45]. Their settlement system consisted of microregions functioning in the same time horizon. Such microregions comprised of at least a single settlement form but most often there were several (two or three) and in some cases up to over a dozen sites of different sizes (larger and smaller stable settlements, campsites) and sometimes a cemetery as well. A typical settlement unit consisted of a homestead, i.e. a house with a yard, covering from several dozen to approx. 200 m^2, standing at a certain distance from each other. At some sites located both in uplands (e.g. the loess part of the Małopolska Upland) and lowlands (Wielkopolska-Kujawy Lowland), more complex settlement patterns were observed, with houses arranged in the shape of an ellipse and forming larger stable settlements. The largest of the latter are estimated to be inhabited by 80-100 people permanently for even 300-400 years [44,46].

The food economy of the TC-associated societies relied on both crop cultivation and animal husbandry. The former is evidenced in pollen profiles and by few charred grains found on settlements [44,46]. The latter played the main role, both in the lowland and upland areas. This is confirmed by grazing markers visible in pollen profiles. Ruminants like cattle and sheep/goat dominated in the structure of herds. Pigs and other domesticates were less important. Overtime the role of cultivation increased but locally it was supplemented with hunting, fishing and gathering.

Funeral ritual of the TC-associated communities was very diversified and sophisticated. It included burying the dead in both under burial mounds and in flat graves. Inhumation and cremation are known along with biritual burials. Occasionally bodies were partially burned and dismembered. Deceased were buried individually or collectively, with preserved anatomical order or without it. Animals were buried as well, separately or with humans.

Collective graves are a characteristic form of TC burials. Radiocarbon dated individuals from over 20 necropolises in regions of Kuyavia, Mazovia, Greater Poland and Lublin Upland allow to estimate the usage of this type of construction for a period from tens to a maximum of 200-300 years [14]. They could therefore be used by several to even dozen of generations. The dead

were placed with their heads against the shorter sides of the burial pit and legs stretched towards its middle. Collective graves contained from two to 30 individuals of both sexes and all age categories [44,46–49].

Altogether, we sampled bones from 150 individuals for DNA extraction. In the end, we obtained nuclear genomes from 68 individuals that are described below.

Cemeteries, graves, and individuals of the Trzciniec culture

Bocheniec, site 2, Jędrzejów district, Małopolska province

The cemetery in Bocheniec is located on Przedborsko-Małogoski Ridge (a series of limestone hills), part of Małopolska Upland, on a sandy terrace on the right bank of the Łośna River, near its estuary to the Nida River. It was excavated in 1959, 1975-1982 and 1984. In its oldest part three collective skeletal graves were found, with funeral ritual and pottery indicating that they may come from the transitional horizon between TC and Lusatian culture [50–52].

poz506 and poz507: Grave 118 was a collective burial with the bottom and walls of the burial pit were lined with stones. It was oriented along the NE-SW axis and measured 3.55-3.65 × 1.20-1.60 m. At its bottom, 10 complete or partially preserved human skulls were found. Seven of them were placed next to the south wall. Bones of the postcranial skeletons were displaced, and their number was smaller than the number of skulls. Probably some of the bones were not preserved [51]. The grave is dated to the declining phase of the TC or its transitional phase to the Lusatian culture [50,53]. Individuals poz506 and poz507 were AMS radiocarbon dated to 1389-1125 BCE (Poz-89341, 3010 ± 35 BP) and to 1411-1217 BCE (Poz-89342, 3050 ± 35 BP), respectively [14]. For DNA extraction we sampled the petrous parts of temporal bones from both individuals. Our genetic results show that individuals poz506 and poz507 were males who belonged to mtDNA haplogroup J1b1a1 and V1a1b, respectively. Poz507 was assigned to Y-chromosomal haplogroup I2a1a1b1a~.

Brodzica, site 19, Hrubieszów district, Lublin Province

This is a multicultural site located in in the Hrubieszów Basin, part of Volhyn Upland, on the southern slope of the Huczwa River valley, on a small loess headland. In 2007, excavations a collective TC burial (no. 27) [54–56].

poz552, poz554, poz555: Grave 27 is interpreted to be the remains of a rectangular mortuary house with a vestibule, measuring 3.40 × 2.10 × 0.40 m. Inside, a collective grave was recorded in which four individuals lay buried on their sides with legs drawn up. In its eastern part, two adult women had been buried back to back (individuals A – poz552 and B – both *Adultus*), with their heads pointing E. In the western part, an adult male (individual D, poz555 – *Maturus*) and a child (individual C, poz554 – *Infans I*) had been placed in parallel with their heads pointing W. The left temporal bone of the child had a greenish hue – a trace possibly left by a decomposed metal (copper? bronze?) ornament. No other grave goods were found. The funerary ritual is characteristic of TC communities and its archaeological dating is justified by two AMS radiocarbon dates obtained for individual poz552: 1499-1311 BCE (Poz-83832, 3145 ± 30 BP) and previously for individual poz554: 1495-1294 BCE (Poz-107536, 3125 ± 30 BP) [27]. For DNA extraction, we sampled petrous parts of temporal bones from individuals poz552 and poz554, as well as tooth from individual poz555. As in our previous study [27], individuals poz554 and poz555 were found to belong to mtDNA haplogroups I1a1a and J1c2, respectively. Our genetic results indicate that individual poz552 carried mtDNA haplogroup I1a1a. Additionally we established individual poz554 to be a male assigned to Y-chromosomal haplogroup R1a1a1~. We also show that individual poz552 was a female and individual poz555 was a male. The three individuals were found to be all sharing 1st degree kinship with each other. The fact that two of them, poz552 and poz554, shared the same mitochondrial haplogroup (I1a1a) and poz555 were assigned to different one (J1c2) was interpreted as the latter being the father of the first two. This was also confirmed by poz552/poz554 sharing relatively lower k1 value then poz555/poz554 and poz555/poz552 in NGSrelate results. Notably the fourth individual, determined based on anthropological analysis to be and adult female, although did not yield enough autosomal data for direct kinship estimation, was determined to belong to the same mt haplogroup as the two children indicating to be their mother, or possibly third sibling[27].

Dacharzów, site 1, Sandomierz district, Świętokrzyskie province

The cemetery is located in the northeast of the Sandomierz Upland, on a loess elevation in the Opatówka River valley [57]. The cemetery centre was occupied by a barrow, covering two centrally-located stone-timber graves in the type of mortuary houses oriented along the NW-SE axis. The structures, built of limestone slabs and blocks as well as pebbles, adjoined each other along their longer sides. The larger one (feature 1a) held six inhumations furnished with bronze ornaments, pottery vessels and animal bones. In the smaller one (feature 1b) cremated

remains of an adult male were buried together with a bronze ear band. In both graves, the human remains had been placed (1a) or spread (1b) on wooden floors.
Eight poorly preserved single (graves 5, 8, 9, 11, 12, 14) or collective burials (grave 2 – double, grave 10 – of four children) were located along the circumference of the barrow. All the graves had stone structures (pavements, cists). Altogether, they contained the remains of 12 individuals: four adults (female and male aged *Adultus-Maturus*) and eight children (*Infans I, Infans II*). Out of the graves located outside the barrow, only grave 2 contained rich grave goods (eight pottery vessels, a glass bead, animal bones). In the other graves, fragments of single vessels (graves 5 and 11) and animal bones (graves 5, 8, 10, 12) were found [57].

poz583: Grave 10 was located on the east side of the barrow. In an oval pit measuring 0.90 × 1.10 × 0.30 m, encircled by an incomplete stone cist, the remains of four children were found. The arrangement of two older ones (*Infans II*) was clear: they were placed on their right and left sides, with legs drawn up and heads pointing SW. Amid the bones of these skeletons, single bones of children aged *Infans I* were identified. The DNA of one individual was analysed: a child who died in its first year of life (10c, poz583) [57]. Individual poz583 was AMS radiocarbon dated to 1499-1299 BCE (Poz-83836, 3135 ± 35 BP) [27]. Petrous part of temporal bone was collected for DNA extraction. Genetic results corroborate with our previous findings [27] and confirm that individual poz583 belong to mtDNA haplogroup W1c. In this study we show that this individual was a male assigned to Y-chromosomal haplogroup R1a~.

poz584: Grave 11 was located on the east side of the barrow and contained the remains of an adult individual. The skeleton was placed in a crouched position on the right side, with the head pointing S. At its feet, fragments of a ceramic vessel were found [57]. It was AMS radiocarbon dated to 1425-1270 BCE (Poz-83837, 3090 ± 30 BP) [27]. The petrous part of a temporal bone was sampled for DNA extraction. As in the previous study [27], individual poz584 was assigned to mtDNA haplogroup V1a1b. In this study we show that this was a female.

Guciów, site 6, Zamość District, Lublin Province, Poland

A multicultural site located in Roztocze Upland, on the sandy terrace of the Wieprz River. TC burial mounds covered cremations with exception of a skeleton burial in barrow XIII.

poz930: The burial pit was located under the central part of the mound. It measured 2.80 × 1.70 m and was partially enclosed with wooden frame. Several posts were distinguished inside,

which probably supported the roof over the grave. Five skulls were found in the bottom layer. Four of them were stacked one inside the other. One had a flint arrowhead found inside the cranium. Below them, arranged diagonally or "crosswise", were the long bones of the upper and lower limbs. Nearby, to the south of them, there was a small pile of fragmented ribs, vertebrae and other bones. The same pile of blades and other bones was visible from the north side. Next to the grave, on the original ground surface, a large post structure was distinguished, interpreted as mortuary house. It had a slightly trapezoidal shape 5.80 × 2.70 × 3.30 m in size [58]. Individual poz930 was AMS radiocarbon dated to 1519-1321 BCE (Poz-107323, 3180 ± 40 BP) [14]. The petrous part of a temporal bone was sampled for DNA extraction. In this study we assigned individual poz930 to be a female that belonged to mtDNA haplogroup V7a.

Gustorzyn, site 1, Włocławek District, Kujawy-Pomorze Province, Poland

The cemetery is located in the Kuyavia region, on a promontory of a flat moraine plateau, separated from the south by the Zgłowiączka river valley and from the west and north by a small valley of an unnamed watercourse disemboguing to Zgłowiączka. Two collective graves (nos. 4 and 5) were discovered during rescue excavations in 1981 on the north-western outskirts of an active gravel pit [59]. In total at least 32 people were buried here but obviously the original number of individuals was higher [44,51,52,60]. We collected petrous part of a temporal bone from 22 individuals for DNA extraction. In the end, we obtained genomic results for 6 individuals.

poz687, poz690, poz693 and poz694: Grave 4 was destroyed in the southern part by a gravel pit. It contained at least 10 individuals (three *Infans*, two *Iuvenis*, three males, two adult individuals without specified sex) [51,60]. Three of the analysed individuals were AMS radiocarbon dated in this study to 1421-1226 BCE (poz687; Poz-142716, 3070 ± 35 BP), 1423-1230 BCE (poz690; Poz-142717, 3075 ± 35 BP), 1416-1226 BCE (poz694; Poz-142718, 3065 ± 35 BP). We sampled petrous parts of temporal bones for DNA extraction. In this study we show that individuals poz690, poz693 and poz694 were males assigned to mtDNA haplogroups K1e, H10a1 and H, respectively, as well as to Y-chromosomal haplogroups I2a1b1a1b1~, I2a1a1b1a1~ and I2a1a1b1a~, respectively. Our results show that poz687 was a female carrying mtDNA haplogroup X2b4.

poz700 and poz702: Grave 5, with a burial pit similar in shape to a rectangle with rounded corners (2.8 × 0.7-0.8 m in size), was surrounded and partially covered with a structure made of boulders of various sizes (covering an area of approx. 3.6 × 2.1 m) [60]. The remains of at least

22 individuals (seven *Infans*, six females, seven males, two adults with no specified sex) were recorded inside the burial pit [51,52]. The deceased were positioned antipodally (antithetically). Skulls were placed at the narrower ends of the burial pit, long bones facing the centre of the grave. Females and children were placed close to each other. In the centre of the pit the remains of a male and a child were discovered (the last individual, "sealing" the grave). Most of the skeletons were lying with their hands close to their heads. Individuals poz700 and poz702 were AMS radiocarbon dated to 1412-1213 BCE (Poz-101025, 3045 ± 35 BP) and 1505-1315 BCE (Poz-101465, 3160 ± 35 BP), respectively [14,61]. We collected petrous parts of a temporal bones for DNA extraction. Genetic results show individual poz700 to be a male and poz702 to be a female. These individuals belonged to mtDNA haplogroups H27 (poz700) and H3g4 (poz702). Individual poz700 was assigned to Y-chromosomal haplogroup I2a1a1b1a1~.

Karczyn, site 21/22, Inowrocław District, Kujawy-Pomorze Province, Poland

The site is located in Kuyavia region, at the edge and on the slope of the Bachorza River valley. The cemetery comprises only six graves, yet the specific spatial arrangement suggests that there might be some burials that remain undiscovered [62].

poz498: Grave no. 200 had a quadrilateral shape 1.74 × 0.48 m in size, aligned along the N-S axis. Two individuals were placed in an alternating arrangement, with their heads towards the north and south, both facing east, equipped with bronze pins [62]. Individual poz498 was AMS radiocarbon dated in this study to 1396-1131 BCE (Poz-85598, 3025 ± 30 BP). The petrous part of a temporal bone was collected for DNA extraction. Our results show that poz498 was a male who belong to mtDNA haplogroup J2b1a6 and Y-chromosomal haplogroup I2a1b2a.

Koszyce, site 3, Proszowice district, Małopolska province, Poland

This multicultural site is located in the Proszowice Plateau, part of Małopolska Upland, on the terrace of the Szreniawa River, where it flows into the Vistula [63]. Within the settlement of the TC inhumation graves were recorded as well. The dead were buried without any grave goods in storage pits. In one case, incomplete human remains were found along with animal skeletons [64,65].

poz543: Grave 151 had a circular shape 1.30 m in diameter. On its bottom, an incomplete skeleton of a child was found. It was AMS radiocarbon dated to 1435-1261 BCE (Poz-83764, 3090 ± 35 BP) [27]. We sampled the petrous part of a temporal bone for DNA extraction. As in previous study [27], individual poz543 was found to belong to mtDNA haplogroup W6a. Our genetic results show that this was a male assigned to Y-chromosomal haplogroup I2a1a1b1a1~.

poz545: Feature 235 was a collective burial. It had a circular shape with a diameter of 2.40 m, and contained four complete, anatomically arranged human skeletons and one loose skull. In addition, a sheep/goat skeleton, cattle bones, and fragments of pottery vessels were found in the burial pit. Individual poz545 was AMS radiocarbon dated in this study to 1391-1130 BCE (Poz-24851, 3020 ± 30 BP). The petrous part of a temporal bone was collected for DNA extraction. This study show that this was a female that carried mtDNA haplogroup K1b2a2.

Nowa Huta Mogiła, site 55, Kraków district, Małopolska province

A multicultural site located on the Proszowice Plateau, part of Małopolska Upland, in the back of the Vistula's terrace, at the confluence with Dłubnia. Burials were found in settlement pits with no grave goods [65–68].

poz551: Grave 228 was a settlement pit secondarily used as a grave. It had an oval shape 3.70 × 3.20 m in size. Its bottom was clearly lower in its eastern parts. In this depression, a skeleton of a child was found, in a supine position with legs flexed and head pointing NW [66]. It was was AMS radiocarbon dated to 1385-1116 BCE (Poz-83765, 2995 ± 35 BP) [27,61]. We sampled the petrous part of a temporal bone for DNA extraction. As in previous study [27], we confirm that this individual carried mtDNA haplogroup T2b. In this study we assigned individual poz551 as a female.

Nowa Huta-Cło, site 65, Kraków District, Małopolska Province, Poland

A multicultural site located on the Proszowice Plateau, part of Małopolska Upland, on a headland of the Vistula's terrace. It included settlement features and a collective grave of the TC, with pottery typical of its classical phase [49,69].

poz155: Grave 108 had an oval shape 3.00 × 3.20 m in size [49,65,69]. On the bottom of the pit, skeletal remains of two young individuals were found. Skeleton 2 (poz155) laid in the eastern part of the pit, in a contracted position on the right side, oriented along the SW-NE axis, with head pointing SW. Next to it, three pottery vessels were found. Skeleton 3 laid in the centre of the pit in a crouched position, on its left side, oriented along the N-S axis, with head pointing N. North of its skull, a pottery vessel was found. Above the two individuals, skeleton of an adult male was found. Only vertebrae, pelvis and bones of lower limbs were articulated. Individual poz155 was AMS radiocarbon dated to 1744-1541 BCE (Poz-83829, 3370 ± 35 BP) [27,61]. We sampled a tooth for DNA extraction. As in previous study [27], we show that this individual to belong to mtDNA haplogroup U5b1b1+16192. In this study we we show that this teenager was a female.

Pielgrzymowice, site 9, Kraków district, Małopolska province, Poland

A multicultural site located on the Miechów Upland, part of the Małopolska Upland, at Dłubnia River, a left-bank tributary of Vistula. Settlement features found on the site yielded materials characteristic of the entire time-span of the TC. Moreover, the site was found to comprise several single and collective TC inhumations in settlement pits with no grave goods [14,65]. Teeth or petrous part of a temporal bones were collected from 18 individuals for aDNA studies. Eventually, genomic results were obtained for the following 11 individuals.

poz715: Feature 66 was a circular pit about 1 m in diameter. On its bottom a complete skeleton of an adult male (poz715) was unearthed. The deceased laid in a crouched position on the left side, along NW-SE axis. Over the head, a flat stone was found. Few pottery shards were found in the pit. Basing on typochronology this individual was dated to 1450-1200 BCE. The petrous part of a temporal bone was collected for DNA extraction. As in previous analyses [27], we confirm this individual to belong to mtDNA haplogroup W3a1. Our results show poz715 to be a male assigned to Y-chromosomal haplogroup I2a1a1b1a1~.

poz713: Feature 213 was an oval pit. In its eastern part, a poorly preserved skeleton of an adult female (*Maturus*) (poz713) was unearthed, lying most likely in the crouched position on the left side with bent legs, almost along the E-W axis, with head pointing E. The pit contained few

pottery shards. Individual poz713 was AMS radiocarbon dated to 1412-1213 BCE (Poz-104949, 3045 ± 35 BP) [27,61]. The petrous part of a temporal bone was sampled for DNA extraction. As in the previous analyses [27], we confirm individual poz713 to belong to mtDNA haplogroup U3a1b. Our results show this individual to be a female.

poz710: Feature 443 was an oval pit, about 1.60 m in diameter. On its bottom, in the southern part, a human skeleton was found, lying in a contracted on the right side with head pointing E. The pit contained few pottery shards. Basing on typochronology this individual was dated to 1450-1300 BCE. The petrous part of a temporal bone was sampled for DNA extraction. As in previous analyses [27], we confirm individual poz710 to belong to mtDNA haplogroup H11. Our results show this individual to be a female.

poz711: Feature 631 was a barely discernible pit. On its bottom, a skeleton of an adult individual (*Senilis*) (poz713) was found, lying on the left side along the NW-SE axis, with stretched legs and head pointing NW. The pit contained few pottery shards. Individual poz713 was AMS radiocarbon dated to 1531-1412 BCE (Poz-104939, 3200 ± 35 BP) [27,61]. The petrous part of a temporal bone was sampled for DNA extraction. As in previous analyses [27], in this study we show individual poz711 to belong to mtDNA haplogroup K1c1. Our results show this individual was a male assigned to Y-chromosomal haplogroup R1a1a1b1a2b3a1d~.

poz712, poz716, poz717, poz719, poz720: Feature 669 was a circular pit 1.50 m in diameter. At a depth of 1 m from the level of discovery, a collective grave was found in which articulated bones of six individuals overlapped. One individual was lying in an extended supine position. Remaining five individuals had been placed on the right or left side, with the legs pulled up to various degrees. The pit contained few pottery shards. Four individuals were AMS radiocarbon dated to 1423-1230 BCE (poz712; Poz-101473, 3075 ± 35 BP), 1497-1302 BCE (poz716; Poz-104943, 3135 ± 30 BP), 1409-1134 BCE (poz717, Poz-104944, 3035 ± 35 BP), 1436-1264 BCE (poz719, Poz-101474, 3095 ± 35 BP), 1405-1131 BCE (poz720, Poz-101475, 3030 ± 35 BP) [27,61]. From these individuals we collected the petrous parts of temporal bones for DNA extraction. Genomes were generated for five skeletons including individuals poz712 (an adult male, *Maturus*), poz716 (an adult female, *Maturus/Senilis*), poz717 (an adult male, *Maturus*), poz719 (an adult male, *Adultus/Maturus*) and poz720 (an adult male, *Adultus/Maturus*). As in previous study [27], we show individuals poz712, poz716, poz717, poz719 and poz720 to belong to mtDNA haplogroup K1a2c, U5b3b1, U5b2a2a, U5b3b1, K1b1a, respectively. In this study individual poz716 was assigned as a female and the rest of the individuals were males carrying

Y-chromosomal haplogroups: R1b1a1b (poz712), I2a(1b1a1b~) (poz717), I2a1b1a1b1~ (poz719), I2a1b2a (poz720). poz.

poz709, poz714: Feature 748 was circular pit 1.50 m in diameter. At its bottom two skeletons were unearthed. An adult female (*Senilis*) (poz714), laid in the western part of the pit in slightly crouched position on the left side, along the N-S axis and with the head pointing N. The skeleton of an adult female (*Maturus*) (poz709) was found in the centre of the pit. Its arrangement suggested that the deceased could have been thrown into the pit. Originally, the body must have been unnaturally twisted as evidenced by the positions of skull, and the bones of legs and arms. The pit contained few pottery shards. Individuals poz709 and poz714 were AMS radiocarbon dated to 1687-1507 BCE (Poz-104940 3320 ± 35 BP) and 1497-1294 BCE (Poz-107546, 3130 ± 35 BP), respectively [27,61]. From both individuals, the petrous parts of a temporal bones were collected for DNA extraction. As in previous analyses [27], in this study we confirm that individuals poz709 and poz714 belonged to mtDNA haplogroup K1b2a2 and U5b2b1a1, respectively. Our genetic results show both individuals to be unrelated females.

Słonowice, site 5 (G), Kazimierza Wielka district, Świetokrzyskie province, Poland

A multicultural site located in western Małopolska, at the southern slopes of an upland sloping towards Małoszówka River, the right-bank of Nidzica – the tributary of the Systematic excavations started in 1979, between 2012 and 2013 also rescue excavations were carried out, preceding the construction of a dam on Małoszowka River [70]. During the rescue excavations a settlement and graves of the TC were discovered. For DNA studies we collected bones from six individuals and obtained genomic results for one of them.

poz833: Grave 147 was cut across in part by a shallow ditch encircling the settlement. It contained a skeleton of a female aged *Maturus* (35-45 years) (poz833), lying in a contracted position on the left side, along the N-S axis with a minor deviation towards NE-SW. The grave was furnished with two pottery vessels. A small fragment of an unidentified bronze ornament was found on the skeleton [70]. This individual was AMS radiocarbon dated in this study to 1873-1622 BCE (Poz-101477, 3420 ± 30 BP). The petrous part of a temporal bone was sampled for DNA extraction. In this study we assigned individual poz833 to be a female carrying mtDNA haplogroup H49.

Żerniki Górne, site 1, Kraków District, Małopolska Province

The site is located on the southern edge of the loess plateau, falling towards the valley. The cemetery was established in the place of the older necropolis of Corded Ware culture (Late Neolithic) and MC (Early Bronze Age) [22,44,71]. At the top and partly on the slopes of the hill, four circles of stones (mainly marl deposits) with a diameter of approx. 4.0 to approx. 6.0 m have been laid. Near them, five pits filled with burnt animal bones and fragments of vessels were unearthed. A thin charred layer covered a considerable part of the hill. The graves of the TC were located to the south and east of the stone circles. In total, 15 collective graves were discovered (nos. 3, 5, 10, 12, 54, 62, 69, 71, 72, 73, 86, 98, 99, 102, 117) and one possible single grave (no 123a). In total ca. 170 people were buried in these graves. Some graves were covered by the mound, some were sunk into it or were placed outside the barrow, without disturbing the mound. The excavation documentation available for the site did not allow as to match the sampled individuals with their position within the collective burials only to the burials themself.

poz649, poz650, poz651, poz652: Grave 10. The burial pit had an outline close to rectangular, 4.0 × 0.75 m in size. It contained poorly preserved remains of ca. 10 individuals. In the eastern part of the grave, five skulls were discovered, in the western part one complete and fragments of the remaining ones were found. Bones of postcranial skeletons were mostly disarticulated, intermingled and mixed inside the chamber. The dead were buried in strongly flexed position. In the eastern part of the grave a single amber bead was found [22]. Four individuals were AMS dated to 1622-1452 BCE (poz650; Poz-107538, 3275 ± 35 BP), 1531-1416 BCE (poz651; Poz-92372, 3205 ± 35 BP), 1598-1416 BCE (poz652, Poz-107539, 3220 ± 35 BP) [27,61]. Basing on typochronology individual poz649 was dated to 1600-1450 BCE. From all these five individuals we collected petrous parts of temporal bones for DNA extraction. As in previous study [27], we confirm that individuals poz649, poz650, poz651, poz652 belonged to mtDNA haplogroups U5a1j, U5b1e1, H11b, U5b2b, respectively. In this study we established that individuals poz650 and poz652 were females, while poz649 and poz651 were males carrying Y-chromosomal haplogroups I2a1a1b~ and I2a(1a1b1a1~), respectively. Individuals poz651, and poz649 were found to share 1st degree kinship. Based on the fact that they were both males assigned to different mitochondrial haplogroups they were determined to be a father-son pair. The two individuals were also found to be both related to poz655 and poz658 from graves 12 and 54 respectively, po651 shared 1st degree and poz649 2nd degree kinship with both individuals. Given the mitochondrial haplogroups of the poz655 and poz658 and the fact that they themselves shared 2nd degree kinship, they were determined to be son of poz651 and half-

sibling of poz649 (in the case of poz658) and full sibling of poz651 and uncle of poz649 (in case of poz655).

poz654, poz655: Grave 12. The burial pit had a regular shape, with W-E orientation, approx. 2.5 × 0.8 m in size. Inside the pit the remains of a wooden coffin with a rectangular outline were found. Three stones were placed between the coffin and the walls of the pit. At least eight individuals were buried in the grave. Four human skulls were found in the eastern part and four in the western part. Bones of postcranial skeletons were mostly disarticulated, intermingled and mixed inside the chamber. In the eastern part of the grave, inside the coffin, a small pottery vessel and an amber bead were unearthed. In the southern part of the grave a bronze pin was found [22]. Individual poz655 was AMS radiocarbon dated to 1498-1306 BCE (Poz-92373, 3140 ± 30BP) [27,61], while individual poz654 was dated to 1500-1300 BCE basing on typochronology. From both individuals we collected the petrous parts of temporal bones for DNA extraction. As in previous study [27], we found that individuals poz654 and poz655 belonged to mtDNA haplogroups T2b and H11b, respectively. In this study we show that individual poz654 was a female and poz655 was a male carrying Y-chromosomal haplogroup I2a1a1b1a1~. The individual poz655 was found to be a brother of individual poz651 from grave 10, and paternal uncle of poz649 and poz658 from graves 10 and 54 respectively (see the description of grave 10 for more details on the pedigree reconstruction).

poz658: Grave 54 was situated 0.5 m south of the grave 10. Upper part of the burial pit was partly destroyed, hence no clear boundary of the grave was recorded. In total 10 individuals were found inside: four females, three males and three children. Eastern part of the grave was better preserved and three skulls and many post-cranial bones were found at its end. More skulls were found in the central and western part of the burial pit. Generally, bones were tightly clustered. Half of an amber bead was the only artefact found within the grave [22]. Individual poz658 was AMS radiocarbon dated to 1423-1230 BCE (Poz-104934, 3075 ± 35 BP) [27,61]. The petrous part of a temporal bone was sampled for DNA analyses. As in the previous study [27], we confirm that individual poz658 belonged to mtDNA haplogroup H13a1a1d. In this study we show this individual to be a male assigned to Y-chromosomal haplogroup I2a1a1b1a1~. The individual was found to be a half-brother of individual poz649 from grave 10, son of poz651 and nephew of poz655 from graves 10 and 52 respectively (see the description of grave 10 for more details on the pedigree reconstruction)

poz659, poz660, poz661, poz662, poz663, poz664, poz665: Grave 62. The burial pit had an almost rectangular outline, approx. 3.8 m × 1.2 m in size. Nine people were buried inside. In

the south-eastern part of the grave there were two articulated skeletons lying in the crouched position on their left side, along the axis of the burial pit, with heads to the SE. To the west of the first of them laid a skeleton placed on its right side, along the axis of the pit, but with the head to the NW. To the south of that specimen a skull of an unidentifiable individual was found. In the widened north-western part of the grave, a badly preserved skeleton was unearthed, placed in a crouched position on the left side, with strongly drawn up legs, along the axis of the pit and with head to the NW. In the north-western part of the grave there was a skeleton placed on its left side, along the axis of the pit, with the head pointing NW, with slightly drawn up legs. Additional three skulls impossible to assign to specific postcranial skeletons were also found in the burial pit. Three pottery vessels were found above the skeletons [22]. Five individuals were AMS radiocarbon dated to 1612-1440 BCE (poz663; Poz-93115, 3250 ± 35 BP), 1608-1425 BCE (poz664; Poz-93454, 3235 ± 35 BP), 1598-1416 BCE (poz659; Poz-104812 3220 ± 35 BP), 1507-1323 BCE (poz660; Poz-101468, 3170 ± 35 BP), and 1536-1416 BCE (poz665; Poz-104932, 3215 ± 35 BP) [14,27,61]. Individual poz661 was dated to 1550-1450 BCE basing on typochronology. From all six individuals we collected the petrous parts of temporal bones for DNA extraction. As in previous study [27], we assigned poz659, poz660, poz661 and poz665 to belong to mtDNA haplogroups N1b1a5, N1b1a5, U4a2, U5a1a1+152, respectively. Our genetic results show that individuals poz663 and poz664 belong to mtDNA haplogroups V and J1c3f, respectively. We also show that poz659, poz660, poz663 and poz664 were females, while poz661 and poz665 were males carrying Y-chromosomal haplogroups R1a1~ and I2a1a1b1a1~, respectively. All individuals except poz661 were found to belong to the same kin group sharing 1st and 2nd degree kinship. Based on the fact that poz660 was a sub-adult that shared 1st degree kinship and mt haplogroup with poz559 she was determined to be the daughter of the latter female. Based on the assigned Y and mt haplogroups the trio of individuals poz663, poz665 were determined to be a pair of parents (the first two) both sharing 1st degree kinship with their son poz662. Additionally poz660 were found to share 2nd degree kinship with poz663, poz665 and poz652. Given lack of kinship between this trio and poz659 they were interpreted to be poz660 paternal grandparents (poz663 and poz665) and uncle (poz662).

poz667, poz670, poz671, poz672, poz673, poz674, poz675, poz678, poz679, poz680: Grave 69. The burial pit had an almost rectangular outline, oriented along the NW-SE axis, approx. 4.7 × 1.6 m in size with four stones in its northern part. Human remains laid in a cluster in the middle of the pit in some distance from its walls. A total number of 26 complete or fragmented human skulls were discovered inside. In the northern part of the grave, 15 skulls and a cluster

of bones of postcranial skeletons were found. In the middle part of the grave an articulated skeleton was found in a position on the left side, along the axis of the pit, with head to the NW, with slightly drawn up legs. In the southern part of the grave there were three skulls and various bones of postcranial skeletons. Fragments of pottery vessels were found throughout the burial pit. In the central part of the pit, an amber bead was found, and in the southern part, a fragment of a bone pin was found [22]. Five individuals were AMS radiocarbon dated to 1613-1446 BCE (poz667, Poz-107541, 3255 ± 35 BP), 1509-1416 BCE (poz671; Poz-107542, 3195 ± 30 BP), 1613-1446 BCE (poz672; Poz-107545, 3255 ± 35 BP), 1613-1446 BCE (poz674; Poz-93455, 3255 ± 35 BP), and 1669-1460 BCE (poz678; Poz-93456, 3295 ± 35 BP) . One individual was AMS radiocarbon dated in this study to 1498-1306 BCE (poz670, Poz-142770, 3140 ± 30 BP). Individuals poz673, poz675, poz679 and poz680 were dated to 1600-1400 BCE basing on typochronology. From these individuals we collected the petrous parts of temporal bones for DNA extraction. As in previous study [27], we retrieved the same mtDNA haplogroups for the following individuals: poz667 (J1c1b), poz671 (J1c1b), poz672 (J1c3j), poz673 (U4d2), poz675 (W3a1), poz678 (T2b), poz679 (H7), poz680 (H11). In this study we show that individual poz670 belong to mtDNA haplogroup U5a2a2a. We also show eight individuals to be females (poz670, poz671, poz672, poz673, poz674, poz678, poz679, poz680) and two individuals poz667, poz675 to be males carrying the same Y-chromosomal haplogroup I2a1a1b1a~.

Individuals poz667 and poz671 were found to share 1st degree kinship and since poz671 was a subadult assigned to the same mt haplogroup, they were determined to be siblings. Adult female poz670 were determined to be daughter of poz667 (due to 1st degree kinship, and different mt haplogroup). Additionally, individuals poz682 and poz684 from grave 99 were determined to be 2nd degree relatives of poz667 determined to be his grandchildren, due to lack of kinship with poz671. Moreover, individual poz746 from grave 71 was found to share 2nd degree kinship with both poz667 and poz671. This could be interpreted as either being their paternal (due to most likely same Y haplogroup) grandfather, uncle, or nephew. One other individual, poz675, from the grave 69 was found to be also related (2nd degree kinship) to poz746 however not to other individuals from the same grave indicating it was they distant relative interpreted as either grandson or nephew of poz746. Finally, poz675 shared 2nd degree kinship to poz747 from grave 98. As poz747 was a sub-adult belonging to different mt haplogroup and same Y haplogroup as poz675 that did not share kinship with poz746 he was interpreted to be either grandson or nephew of poz675.

poz745 and poz746: Grave 71. The burial pit with NW-SE orientation had a non-regular, almost oval outline, approx. 4.5 m × 1.5 m in size. At least 17 individuals were buried inside. Human remains laid in a cluster separated by empty space from the walls of the grave. Most of the long bones laid in a non-anatomic order in the northern and central part of the pit. In southern part of the grave a cluster of at least seven skulls (and their fragments) was located. Five skulls and numerous bones of post-cranial skeletons were found in a central part of the pit, and four skulls were lying in north-eastern part of the grave. In north-western part of the burial pit there was only one articulated skeleton lying on its left side, with head pointing NW. The grave was furnished with pottery, an amber bead, bronze ornaments and flint artefacts [22]. Individual poz745 was AMS radiocarbon dated to 1511-1327 BCE (Poz-93457, 3180 ± 35 BP) [14,61]. We sampled the petrous parts of temporal bones for DNA extraction. As in previous analyses [27] our results indicate that individual poz745 belonged to mtDNA haplogroup H11. In this study we indicate individual poz746 to be a male assigned to mtDNA haplogroup H2a1 and Y-chromosomal haplogroup I(2a1a). The individual poz746 was found to share 2nd degree kinship with poz667, poz670 and poz675 from grave 69 (detailed description of the pedigree in the description of grave 69).

poz748: Grave 73. The burial pit, oriented along the NW-SE axis, was almost rectangular in outline, 4.75 m × 1.6 m in size. There were two skeletons in the north-western part of the grave, with their heads pointing NW. In the south-eastern part three skulls were unearthed. NW from them a cluster of mixed bones from incomplete post-cranial skeletons was found. In total at least 11 individuals were buried in the grave. The grave was furnished with pottery and large number of both bronze ornaments and flint artefacts [22]. Individual poz748 was AMS radiocarbon dated to 1598-1416 BCE (Poz-93458, 3220 ± 35 BP) [27,61]. We sampled the petrous part of a temporal bone for DNA extraction. As in the previous study [27], we assigned this individual to mtDNA haplogroup J1c3. Our results show poz748 to be a male who belonged to Y-chromosomal haplogroup I2a1a1b1a~.

poz747: Grave 98. The burial pit was irregular, almost rectangular in outline, oriented along the N-S axis, 4.4 m × 1.5 m in size. The deceased were buried in the central part of the pit. The grave contained at least 21 individuals. In northern part of the pit eight skulls were found and bones of post-cranial skeletons were deposited south from them. Their legs were flexed. Additional cluster of four skulls was found in the middle and southern part of the grave. Another five skulls were distributed in various parts of the grave. Between the human bones, mainly in the vicinity of the skulls, some ornaments made of bronze and amber, and a few pottery shards

were found [22]. Individual poz747 was AMS radiocarbon dated to 1492-1291 BCE (Poz-93459, 3120 ± 30 BP) [27]. The petrous part of a temporal bone was sampled for DNA extraction. As in the previous study [27], we confirm that individual poz747 belonged to mtDNA haplogroup U4b1a1a1. Our results indicate this individual to be a male assigned to Y-chromosomal haplogroup I2a1a1b1a1~. The individual were found to share 2nd degree kinship with poz675 from grave 69.

poz682, poz683, poz684: Grave 99. The burial pit had an almost rectangular outline, NW-SE orientation, and was approx. 3.6 × 0.75 m in size. At least 21 people were buried inside. In the northern part of the grave seven skulls were found, the post-cranial skeletons associated with them lay along the longer axis of the grave, with heads to NW and with strongly drawn up legs. In the south-eastern part of the grave, seven or eight skeletons in the same position were found, with heads to SE. Ceramic vessels as well as ornaments made of bronze, amber, glass, bone and other materials were found between the human bones [22]. Two individuals were AMS radiocarbon dated to 1448-1272 BCE (poz683; Poz-92374, 3110 ± 35 BP) and 1408-1226 BCE (poz684; Poz-92375, 3055 ± 30 BP) [27,61]. Individual poz682 was dated to 1450-1250 BCE basing on typochronology. We collected the petrous parts of temporal bones from a child (poz682) and two adults (poz683 and poz684) for DNA extraction. As in the previous study [27], we assigned mtDNA haplogroups for individuals poz682, poz683 and poz684 to H11b, H49, H11b, respectively. Our results indicate poz682 and poz683 to be females and poz684 to be a male carrying Y-chromosomal haplogroup I2a1a1b1a1~. Individual poz682 and poz684 were found to share 1st degree kinship. As poz682 was a subadult and both individuals were assigned to the same mt haplogroup they were determined to be siblings. Additionally, both of them shared 2nd degree kinship with poz667 from grave 69 determined to be their paternal grandfather (interpretation of this kinship in the description of grave 69).

The Komarów culture

Polish and Ukrainian archaeologists have distinguished many manifestations of the Komarów culture (KC), including the Eastern Trzciniec culture [72], Trzciniec-Komarów culture [73,74], as well as two completely separate archaeological cultures [73], and have even considered them to be a large, interconnected cultural circle [75]. The latest approach in interpreting this cultural phenomenon is to define the KC as a south-eastern part of the TCC [44,76].

The KC covers areas stretching from the basin of the upper Dniester to the Podolia Upland and the southern part of the Volhynian Upland. It is known mainly for extensive barrow cemeteries, with up to several hundred burial mounds, arranged in linear and linear-group forms. There is an extensive funeral architecture under the embankments, including graves in the type of houses of the dead and accompanying features of a ritual character [77,78]. In the southern zone, cremation rituals are more common. In Volhynia and in the so-called Biały Potok group in Podolia inhumation is more popular [79]. Both individual and collective burials are known.

Only a few settlements have been documented, on the upper Dniester and Prut Rivers as well as in Volhynia, in which relics of small buildings of rectangular outline, sometimes with a sunken floor, were discovered [44,73,80]. The KC associated population probably practiced mobile animal husbandry.

Altogether, we sampled bones from seven individuals for DNA extraction. In the end, we obtained nuclear genomes from five individuals that are described below.

Beremiany, site Red Field, Buchach region, Tarnopil Province, Ukraine

The site (currently Beremyani) is situated in the Podolia Upland, on the Dniester River, close to mouth of the Strypa River, in the field known before the Second World War as "Red Field" (currently "Red Hill") [81,82].

poz643, poz644: Grave excavated in 19th c. AD. It represents the Biały Potok group of the KC. It was characterised by a stone construction on the circular plan, inside which two bone deposits were discovered [81,82]. It cannot be explicitly stated how many individuals were buried inside (two or three?). The majority of bone fragments belong to a female (*Adultus*). However, a fragment of the temporal bone, because of its thickness, was attributed to a male. In the third case it was impossible to determine either the sex or whether the examined fragment belonged to the aforementioned male individual. Grave goods included a flint tool, bronze items (a pin and a damaged earring or pendant), and a pottery vessel [80–82]. Individuals poz643 and poz644

were AMS radiocarbon dated to 1919-1701 BCE (Poz-61777, 3500 ± 30 BP) and 1875-1620 BCE (Poz-76313, 3420 ± 35 BP), respectively [14]. We sampled the petrous parts of temporal bones for DNA extraction. In this study we show that individuals poz643 and poz644 belonged to mtDNA haplogroups W6a and U5a1g1, respectively. Our results show that poz644 was a female, while poz643 was a male carrying Y-chromosomal haplogroup I1/I1a2a1a1~.

Biały Potok, Chortkiv district, Tarnopil province, Ukraine

The site is situated on Podolia Upland, at the northern edge of the Młynówka River (now Bilyi Potik) valley, right tributary of the Siret, several kilometres north from its confluence with the Dniester [77,79,83,84]. A Middle Bronze Age necropolis was excavated here before the Second World War. Graves I-II (with two burials) and IV (a cist grave with one burial) were excavated in 1925.

poz296: Grave I-II contained badly damaged skeletal remains of two individuals. The first (female, *Adultus*?) lay in a crouched position, the second (poz296, male, was found next to it. The deceased was placed a flexed position on the right side [83]. Both skeletons have their heads pointing west. Two vessels were found in the grave (an amphora and a bowl) which represent Biały Potok group of the Middle Bronze Age [77]. Individual poz296 was AMS radiocarbon dated to 1615-1447 BCE (Poz-70477, 3260 ± 35 BP) [84]. We sampled the tooth for DNA extraction. As in previous study [27], we confirm this individual to belong to mtDNA haplogroup H49. In this study he was assigned to Y-chromosomal haplogroup R1a1a1~.

Kordyshiv, site VIII, Schumsk District, Tarnopil province, Ukraine

The site is located on a high plateau, on the left bank of the Vilia River in western part of Podolia Upland. At the beginning of the 20th c. AD, more than 20 burial mounds were recorded there. Excavations revealed the presence of three burial mounds belonging to the KC (burial mounds 1 and 3) from the first half of the 2nd millennium BCE and the Corded Ware culture (2nd half of the 3rd millennium BCE) and secondary burials of the KC [85]. Nine burials have been found in the mound 3, including four of the KC.

poz636, poz637: Barrow 3. In grave 1, well-preserved skeletal remains of two females were found. They were richly equipped with bronze items: bracelets, shoulder straps and pins for fastening burial robes, and a clay vessel[85]. Individuals poz636 and poz637 were AMS

radiocarbon dated to 1687-1510 BCE (Poz-83755, 3325 ± 35 BP) and 1686-1504 BCE (Poz-83756, 3315 ± 35 BP), respectively [14,61]. We sampled the petrous parts of temporal bones from both individuals. Our corroborated results show that individuals poz636 and poz637 were females assigned to mtDNA haplogroup X2b10 and U4a2a, respectively.

Influence of the 15k capture on the data analysis

To test the potential biases introduced by the data obtained from libraries that underwent the enrichment procedure several tests were performed. First for the three samples with highest proportion of data originating in enriched libraries, 100 repeats of random selection of 7 million reads, with the use of samtools view -s option were performed. Then the data was mapped to the hs37d5 version of the human reference genome as described in the methods section of the manuscript. Then the genotyping rate and coverage for both 15k SNPs targeted by enrichment and SNPs from HO dataset (after removing SNPs within 600bp windows surrounding the 15k panel) was calculated using samtools coverage tool. The results (Table S1) show that while the enrichment was successful and the number of genotyped targeted SNPs and their average coverage has increased, the genotyping rate and coverage of the SNPs outside of the targeted regions were not significantly impacted by the enrichment process. Therefore, majority of the SNPs used in subsequent analyses were not the SNPs target by the enrichment procedure.

Additionally, we explored the affect the use of capture data could have on population genetics analyses by carrying the PCA analyses separately on the capture and shotgun data. The results show that while there is some bias observed in the sample placement on PCA plot, the observed difference would not lead to changes in the interpretation of the final results, as the individuals are still placed within the general TCC population, no matter which subset of the data is used **(Supplementary Fig. 1A)**. Similarly, we run f3 statistics separately for shotgun, capture and merged datasets in a form of f3(YRI, tested individual, ancient population). The results show that, while in one case (poz660) the result for capture data were outside of the f3 values range for TC population, in all cases the values for the merged data were close to the values acquired for only shotgun sequences **(Supplementary Fig. 1B)**. We therefore argue that using merged shotgun and capture data, does not affect the final interpretation of the results in a meaningful way.

Table S1. The average, among 100 subsamples, number of successfully genotyped autosomal SNPs and their mean coverage. Results shown for SNPs overlapping the enrichment probes (15k) and SNPs from HO panel outside 600bp windows surrounding the targeted SNPs.

library	15k SNP average coverage	15k SNP average SNP count	HO average coverage	HO average SNP count
poz659 capture	6.8940	13 011	0.1673	87 934
poz659 shotgun	0.1651	1 984	0.1619	90 509
poz660 capture	114.0710	13 107	0.2522	65 887
poz660 shotgun	0.1975	2 351	0.1871	103 388
poz665 capture	41.8223	13 100	0.1972	69 497
poz665 shotgun	0.1576	1 909	0.1520	85 421

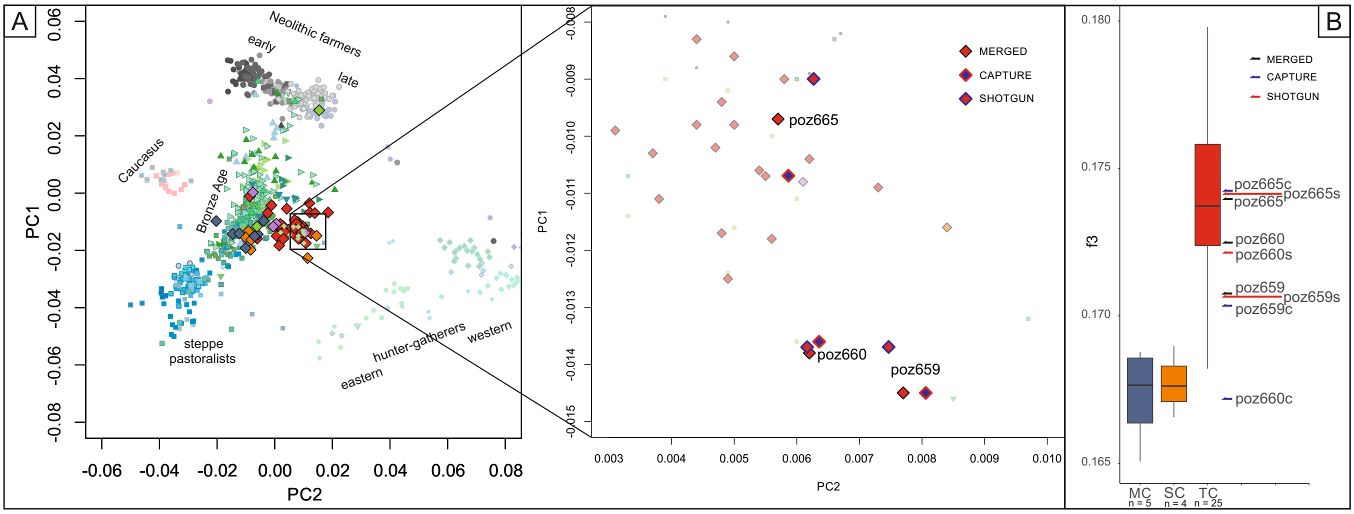

Supplementary Fig. 1. The biases introduced by capture. A PCA plot, right panel shows zoomed in part of a plot from Fig. 1D from the main text of the manuscript. B values for f3 statistics in a form f3(YRI, tested individual, NBL), compared to ranges of values acquired for individuals associated with Mierzanowice (MC), Strzyżów (SC) and Trzciniec (TC) Cultures. The box plot shows the median (middle horizontal line), interquartile range (25th and 75th percentile) indicated with lower (25th percentile) and upper (75th percentile) hinges of the box, and whiskers extending to the lowest (highest) value that is within 1.5 times the interquartile range of the upper (lower) hinge.

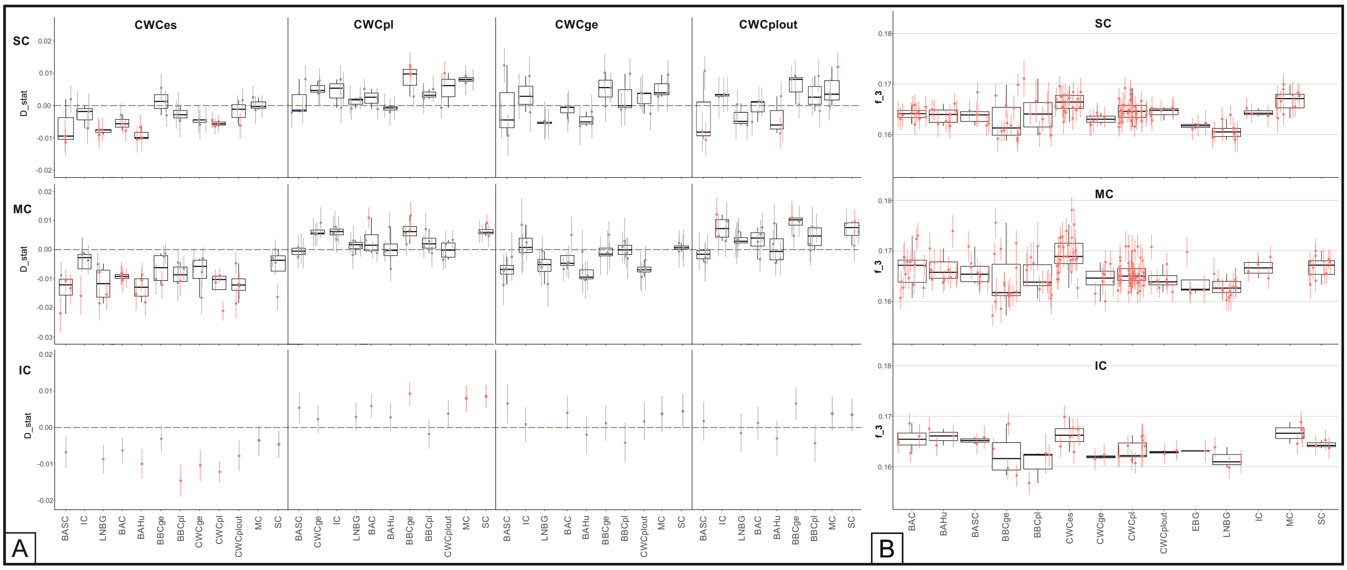

Supplementary Fig. 2. The genetic affinities of Early Bronze Age Populations as estimated by f3 and D statistic. **A** The D statistics in the form of D(YRI, tested individual(left), population 1(top), population 2(bottom). **B** Results of pairwise f3 statistic in the form of f3(YRI, population 1 individual, population 2 individual)

The box plots in all the panels show the median (middle horizontal line), interquartile range (25th and 75th percentile) indicated with lower (25th percentile) and upper (75th percentile) hinges of the box, and whiskers extending to the lowest (highest) value that is within 1.5 times the interquartile range of the upper (lower) hinge. The overlying points display individual values, those for which |Z| >= 2 are in red and the point whiskers represent one standard deviation.

The labels in all panels are as follows: TC – Trzciniec Culture, KC – Komarów Culture, SC – Strzyżów Culture, MC – Mierzanowice Culture, IC – Iwno Culture, BAC – Battle Axe Culture, BAHu – Bronze Age Hungary, BASC – Bronze Age Scandinavia, EBG – Early Bronze Age Germany, LNBG – Late Neolithic/Bronze Age Germany, BBC – Bell Beaker Culture, CWC – Corded Ware Culture.

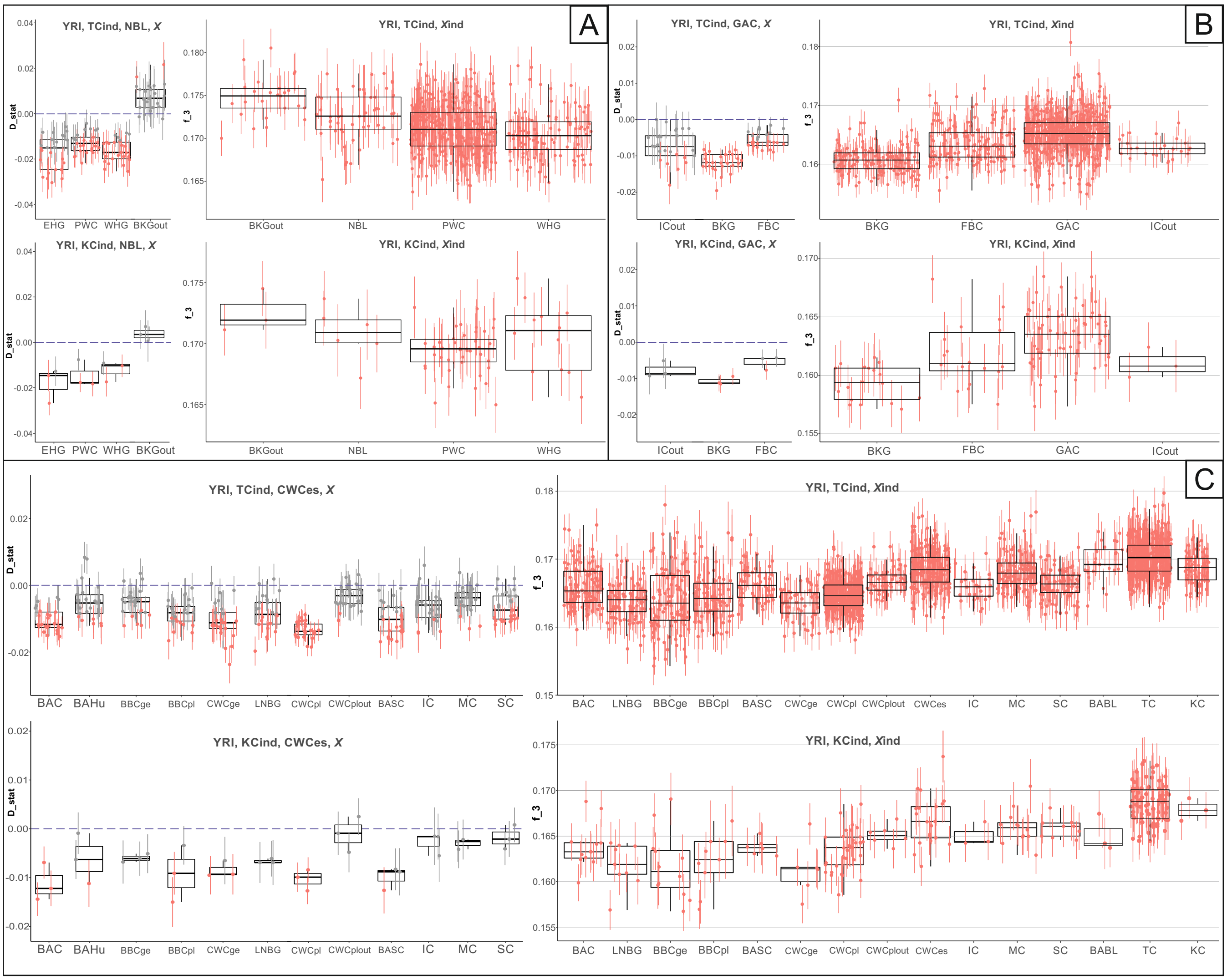

Supplementary Fig. 3. The genetic affinities of Middle Bronze Age Populations as estimated by f3 and D statistic for **A** hunter-gatherer, **B** Neolithic farmer and **C** Early Bronze Age ancestry.
On the left side of each panel the D statistics in the form of D(YRI, tested individual(left), population 1(top), population 2(bottom)) are shown. On the right side the Results of pairwise f3 statistic in the form of f3(YRI, population 1 individual, population 2 individual) are displayed.
The box plots in all the panels show the median (middle horizontal line), interquartile range (25th and 75th percentile) indicated with lower (25th percentile) and upper (75th percentile) hinges of the box, and whiskers extending to the lowest (highest) value that is within 1.5 times the interquartile range of the upper (lower) hinge. The overlying points display individual values, those for which |Z| >= 2 are in red and the point whiskers represent one standard deviation.

The labels in all panels are as follows: TC – Trzciniec Culture, KC – Komarów Culture, SC – Strzyżów Culture, MC – Mierzanowice Culture, IC – Iwno Culture, BABL – Bronze Age Baltic, BAC – Battle Axe Culture, BAHu – Bronze Age Hungary, BASC – Bronze Age Scandinavia, BBC – Bell Beaker Culture, CWC – Corded Ware Culture, BKG – Brzesc Kujawski Group, BKGout - BKG hunter-gatherer, EBG – Early Bronze Age Germany, EHG – Eastern hunter-gatherers, FBC – Funnel Beaker Culture, GAC – Globular Amphora Culture, ICout – IC outlier, LNBG – Late Neolithic/Bronze Age Germany, NBL – Neolithic Baltic hunter-gatherers, PWC – Pitted Ware Culture, WHG – Western hunter-gatherers

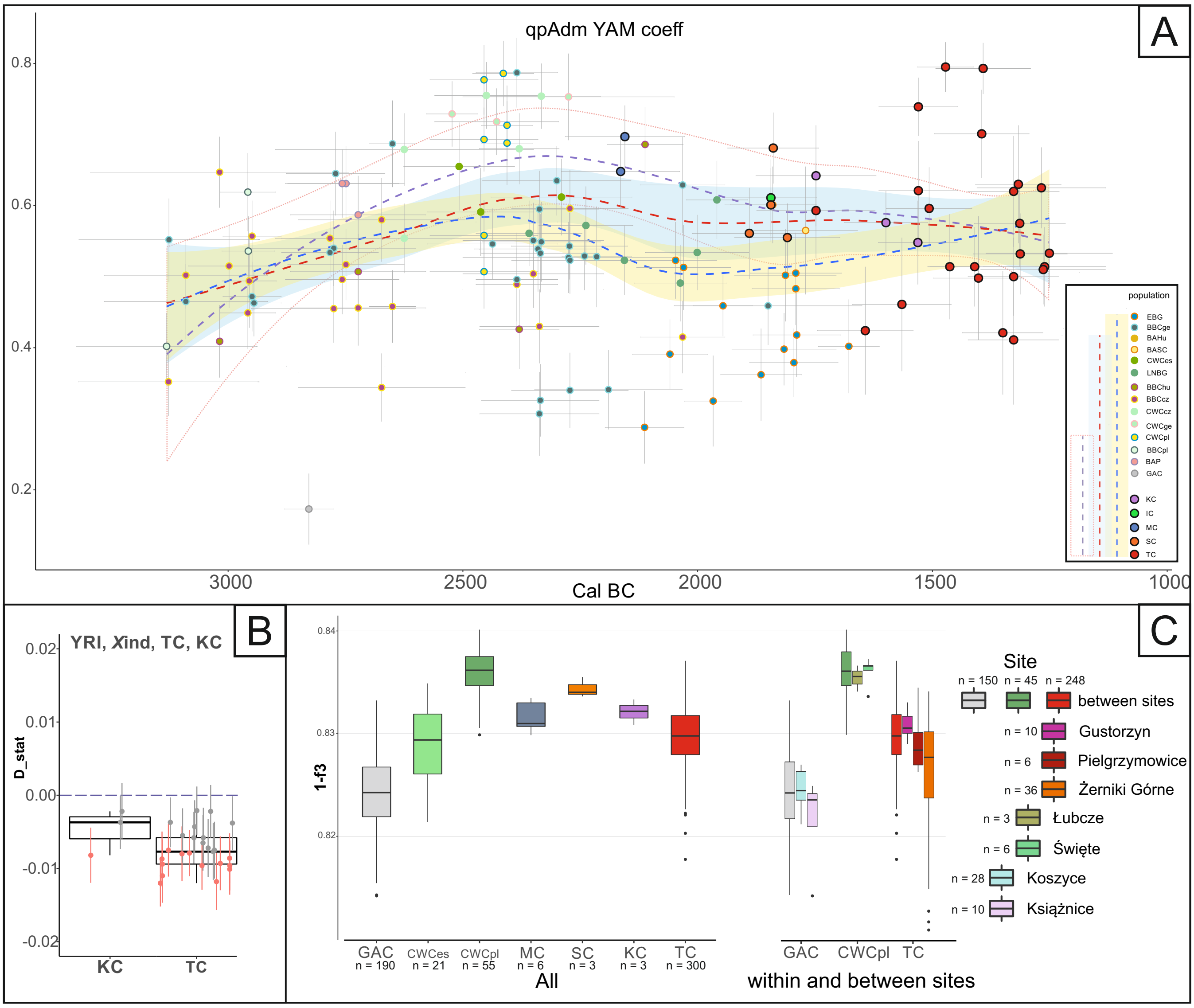

Supplementary Fig. 4. A Amount of steppe ancestry (± one standard error) estimated for new (outlined in black) and reference individuals from the Final Neolithic to the Middle Bronze Age. The degree of ancestry was estimated from three-way admixture models including the WHG, AN and YAM, the points represent coefficient for WHG ancestry calculated using qpAdm (with p value for nested models < 0.05). The dates are shown with 2σ error bars. The fit lines in panels display smoothed conditional means with corresponding 95% confidence intervals for all individual (blue) and after removal of selected populations as shown in the legend of the figure. **B** Results of D statistics in the form of D(YRI, population X individual, TC, KC). The values for which |Z| >= 2 are in red. **C** Genetic diversity as estimated by distribution of shared genetic drift between the individuals within each population for newly published data and two reference populations. Where possible, sites yielding multiple individuals are shown separately.

The box plots in panels B and C are same as those in figures S46 and S47 except for points in panel C which display only the individual values outside of 1.5x interquartile range form the hinges.

The labels in all panels are as follows: IC – Iwno Culture, KC – Komarów Culture, MC – Mierzanowice Culture, SC – Strzyżów Culture, TC – Trzciniec Culture, BAC – Battle Axe Culture, BAHu – Bronze Age Hungary, BAP – Bronze Age Poland, BASC – Bronze Age Scandinavia, BBC – Bell Beaker Culture, BKG – Brzesc Kujawski Group, CWC – Corded Ware Culture, EBG – Early Bronze Age Germany, , GAC – Globular Amphora Culture, LNBG – Late Neolithic/Bronze Age Germany, MNG Middle Neolithic Germany, NBL Neolithic Baltic, NCHu Neolithic/Chalcolithic Hungary.

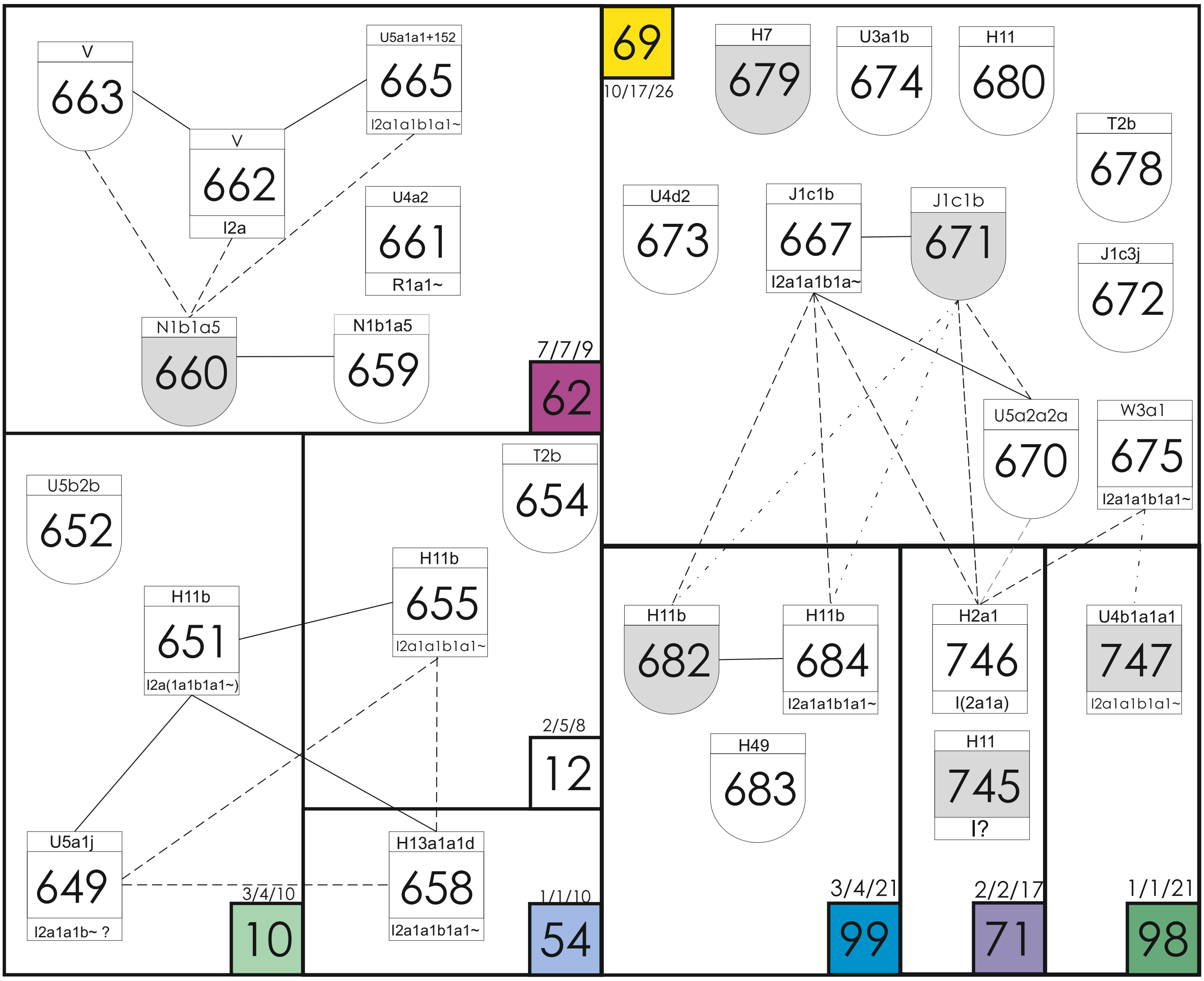

Supplementary Fig. 5. The detected 1st-degree (solid line), 2nd-degree (dashed line), and 2nd/3rd-degree (dot-dashed line) kinship among the individuals buried in Żerniki Górne cemetery. The numbers and colours correspond to the burial chambers in Figure 3. The number above the burial chamber labels describes the number of sampled individuals relative to the minimum number of individuals interred in each chamber in the form of: analysed individuals passing filters/sampled individuals/minimum number of individuals.

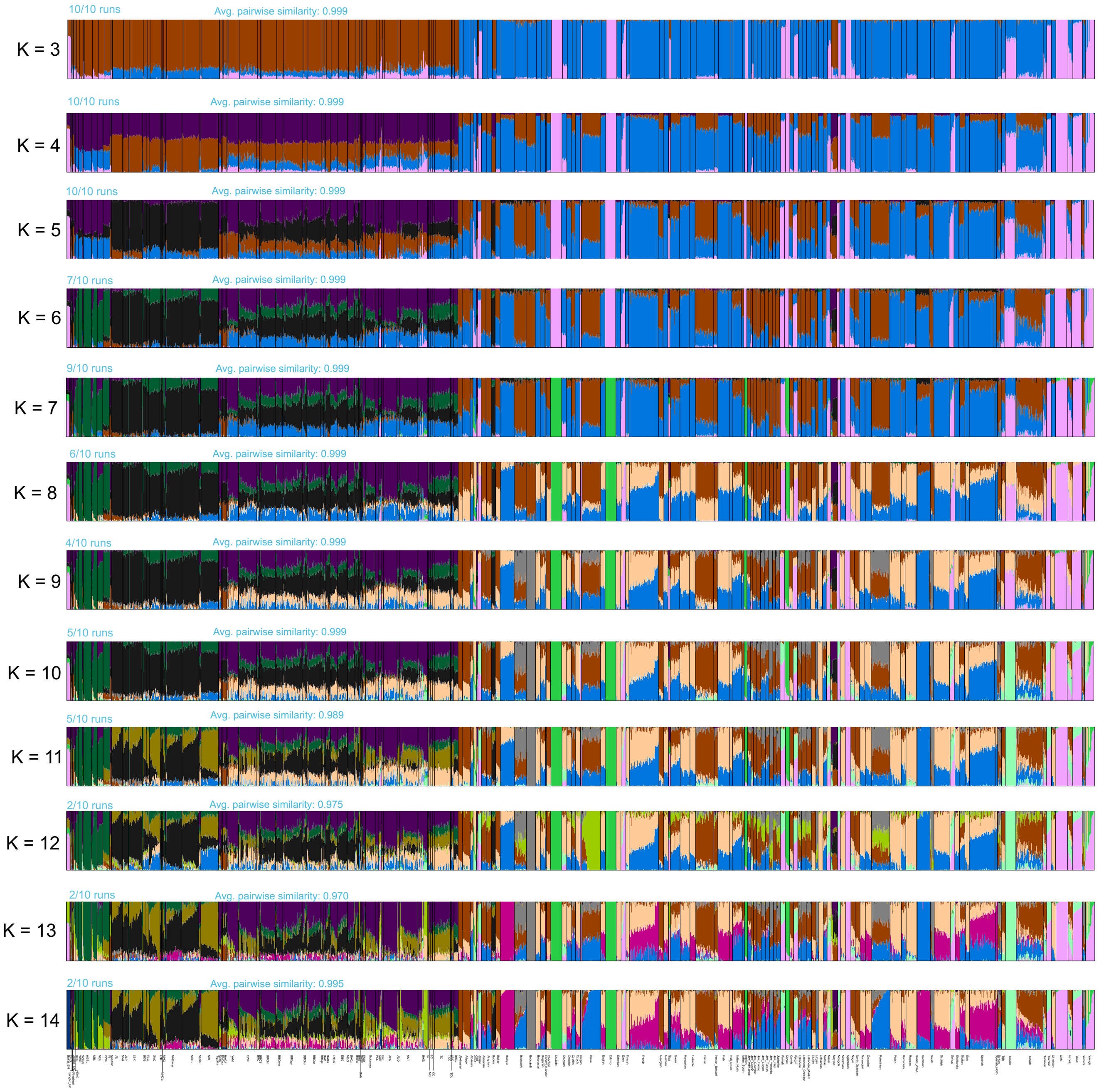

Supplementary Fig. 6. Admixture analysis (K3-K14) results for ancient and modern Eurasian populations. The abbreviations for the ancient populations can be found in Extended Dataset S2.

Supplementary References

1. Kośko, A. *Rozwój kulturowy społeczeństw Kujaw w okresach schyłkowego neolitu i wczesnej epoki brązu.* (Uniwersytet im. Adama Mickiewicza w Poznaniu, 1979).

2. Czebreszuk, J. *Społeczności Kujaw w początkach epoki brązu*. (Uniwersytet im. Adama Mickiewicza w Poznaniu, Poznańskie Towarzystwo Prehistoryczne., 1996).

3. Makarowicz, P. *Rola społeczności kultury iwieńskiej w genezie trzcinieckiego kręgu kulturowego : (2000-1600 BC)*. (Uniwersytet im. Adama Mieckiewicza, 1998).

4. Czebreszuk, J. & Szmyt, M. Background to Beakers. Inquiries into regional cultural backgrounds of the Bell Beaker complex. in *Bell Beakers and the cultural milieu of north European plain* (eds. Fokkens, H. & Nicolis, F.) 157–175 (Sidestone Press, 2012).

5. Czebreszuk, J. *Schyłek neolitu i początki epoki brązu w strefie południowo-zachodniobałtyckiej (III i początki II tys. przed Chr.) : alternatywny model kultury*. (Wydawnictwo Naukowe UAM, 2001).

6. Makarowicz, P. Northern and Southern Bell Beakers in Poland. in *The Northeast Frontier of Bell Beakers.* (eds. Czebreszuk, J. & Szmyt, M.) 137–154 (Archaeopress, 2003).

7. Makarowicz, P. Concerning the Development of the Metallurgy in Early Bronze Age Societies on the Polish Lowlands. *Lietuvos Archeologija* **199**, 221–239 (2000).

8. Grossman, A., Stanaszek, Ł., Kapala, W. & Pospieszny, Ł. Zespół grobów z wczesnej epoki brązu na stanowisku 6 w Gąsawie, woj. kujawsko-pomorskie. in *Vir Bimaris : od kujawskiego matecznika do stepów nadczarnomorskich : studia z dziejów międzymorza bałtycko-pontyjskiego ofiarowane profesorowi Aleksandrowi Kośko* (eds. Szmyt, M., Chachlikowski, P., Czebreszuk, J., Ignaczak, M. & Makarowicz, P.) 71–87 (Instytut Archeologii Uniwersytetu im. Adama Mickiewicza w Poznaniu, 2019).

9. Kośko, A. Z badań nad udziałem tradycji kultury amfor kulistych w sferze światopoglądowo-obrzędowej społeczeństw kultury iwieńskiej. in *Miscellanea archaeologica Thaddaeo Malinowski dedicata / quae Franciscus Rożnowski redigendum curavit* (ed. Rożnowski, F.) 17–22 (Sorus, 1993).

10. Harney, É. *et al.* Ancient DNA from Chalcolithic Israel reveals the role of population mixture in cultural transformation. *Nature Communications* **9**, 3336 (2018).

11. Mathieson, I. *et al.* The genomic history of southeastern Europe. *Nature* **555**, 197–203 (2018).

12. Makarowicz, P. Osadnictwo społeczności kultury iwieńskiej oraz trzcinieckiego kręgu kulturowego (2400-1400/1300 BC). in *Osadnictwo kultur późnoneolitycznych oraz interstadium epok neolitu i brązu: 3900-1400/1300 BC* (eds. Kośko, A. & Gierlach, M.) 457–550 (Wydawnictwo Poznańskie, 2000).

13. Krenz-Niedbała, M. Charakterystyka antropologiczna społeczności kultury ceramiki sznurowej, kultury iwieńskiej oraz trzcinieckiego kręgu kulturowego. in *Osadnictwo kultur*

późnoneolitycznych oraz interstadium epok neolitu i brązu: 3900-1400/1300 BC (eds. Kośko, A. & Gierlach, M.) 563–567 (Wydawnictwo Poznańskie, 2000).

14. Makarowicz, P. *et al.* The absolute chronology of collective burials from the 2nd millennium BC in East Central Europe. *Radiocarbon* **63**, 669–692 (2021).

15. Kadrow, S. *Gospodarka i społeczeństwo : wczesny okres epoki brązu w Małopolsce*. (Instytut Archeologii i Etnologii Polskiej Akademii Nauk, 1995).

16. Kadrow, S. & Machnik, J. *Kultura mierzanowicka. Chronologia, taksonomia i rozwój przestrzenny.* (PAN. Oddział w Krakowie, 1997).

17. Madej, P. & Valde-Nowak, P. *Kultura mierzanowicka w karpackim dorzeczu Wisły.* (Muzeum Podkarpackie w Krośnie : Wydawnictwo Rutheus, 2020).

18. Rydzewski, J. Przemiany stref zasiedlenia na wyżynach lessowych Małopolski w epoce brązu i żelaza. *Archeologia Polski* **31**, 125–194 (1986).

19. Włodarczak, P. Małopolska at the beginning of the Bronze Age (2000-1600 BC). in *The Past societies. Polish lands from the first evidence of human presence to the early Middle Ages. 3. 2000-500 BC* (ed. Bugaj, U.) 275–336 (Instytut Archeologii i Etnologii Polskiej Akademii Nauk, 2017).

20. Machnik, J., Bagińska, J., Koman, W., Libera, J. & Gazda, L. *Neolityczne kurhany na Grzędzie Sokalskiej w świetle badań archeologicznych w latach 1988–2006*. (2009).

21. Kadrow, S. *U progu nowej epoki. Gospodarka i społeczeństwo wczesnego okresu epoki brązu w Europie Środkowej.* (Instytut Archeologii i Etnologii Polskiej Akademii Nauk, 2001).

22. Kempisty, A. *Schyłek neolitu i początek epoki brązu na Wyżynie Małopolskiej w świetle badań nad kopcami*. (Wydawnictwa Uniwersytetu Warszawskiego, 1978).

23. Bąbel, J. *Cmentarzyska społeczności kultury mierzanowickiej na Wyżynie Sandomierskiej*. (Instytut Archeologii Uniwersytetu Rzeszowskiego, 2013).

24. Kadrow, S., Machnik, A. & Machnik, J. *Iwanowice. Stanowisko Babia Góra, część II. Cmentarzysko z wczesnego okresu epoki brązu.* (Instytut Archeologii i Etnologii Polskiej Akademii Nauk, 1992).

25. Baczyńska, B. *Cmentarzysko kultury mierzanowickiej w Szarbi, woj. kieleckie. Studium obrządku pogrzebowego*. (Instytut Archeologii i Etnologii Polskiej Akademii Nauk, 1993).

26. Polańska, M. Badania ratownicze cmentarzysk kultury strzyżowskiej w Hrebennem, woj. zamojskie. *Archeologia Polski Środkowowschodniej* **3**, 78–85 (1998).

27. Juras, A. *et al.* Mitochondrial genomes from Bronze Age Poland reveal genetic continuity from the Late Neolithic and additional genetic affinities with the steppe populations. *American Journal of Physical Anthropology* **172**, 176–188 (2020).

28. Kłosińska, E. M., Libera, J. L., Twardowski, W., Szczurowski, J. & Szostek, K. The presumed Early Bronze Age grave from the Radom Plain. *Spraw. Arch.* **71**, 273–301 (2019).

29. Krauss, A. Cmentarzysko i osada kultury mierzanowickiej w Pieczeniegach, pow. Miechów. *Materiały Archeologiczne* **9**, 159–165 (1967).

30. Krauss, J. & Krauss, A. Cmentarzysko kultury mierzanowickiej w Świniarach Starych, pow. Sandomierz. *Materiały Archeologiczne* **12**, 109–136 (1971).

31. Kłosińska, E., Taras, H. & Sadowski, S. Doslidshennya pamyatok dobi bronzi ta ranogo zaliza v 2000-2007 rokakh u seredniy chastiny schidnoy Polshchi. *Materialy i Doslidžennja z Archeolohii Prykarpattja i Volyni* **14**, 243–257 (2010).

32. Głosik, J. Kultura strzyżowska. *Materiały Starożytne* **11**, 111–163 (1968).

33. Taras, H. Na przedpolu świata pontyjskiego – osadnictwo kultury strzyżowskiej (2000/1950-1600 przed Chr. in *Pradzieje południowo-wschodniej Lubelszczyzny* (ed. Banasiewicz-Szykuła, E.) 85–94 (Wojewódzki Urząd Ochrony Zabytków w Lublinie, 2007).

34. Ślusarski, Z. & Ślusarska-Polańska, M. Badania stanowisk kultury strzyżowskiej w Raciborowicach Kolonii, woj. Chełm, w latach 1956, 1958 i 1959. *Spraw. Arch.* **40**, 167–196 (1988).

35. Bargieł, B. Kultura strzyżowska w świetle znalezisk grobowych. *Wiadomości Archeologiczne* **LVIII**, 65–99 (2006).

36. Hyrchała, A. Kultura Strzyżowska – początek nowej epoki. in *Wojownik i księżniczka. Archeologia - medycyna sądowa – sztuka* 32–51 (Hrubieszów: Muzeum im. ks. St. Staszica, 2015).

37. Polańska, M. Badania na stanowiskach 2 i 4 w Raciborowicach Kolonii, gm. Białopole. in *Informator archeologiczny: badania* vol. 21 40–41 (Narodowy Instytut Dziedzictwa, 1994).

38. Polańska, M. *Hrebenne, gm. Horodło, woj. zamojskie. Sprawozdanie z archeologicznych badań ratowniczych na stanowisku 31*. (unpublished manuscript, 1998).

39. Kozak-Zychman, W. & Pilarska, E. *Analiza antropologiczna materiałów kostnych ze stan. 10, 31 i 34 w miejscowości Hrebenne w woj. zamojskim*. (unpublished manuscript, 1998).

40. Kutyłowska, I. & Kutyłowski, A. Materiały kultury wstęgowej ceramiki malowanej i kultury strzyżowskiej z miejscowości Nieledew, pow. Hrubieszów. *Wiadomości Archeologiczne* **XXXV**, 327–339 (1970).

41. Budziszewski, J. *et al.* Kurhany ze stanowiska 30 w Stryjowie, pow. krasnostawski. in *Schyłek neolitu na Wyżynie Lubelskiej* (eds. Jarosz, P., Libera, J. & Włodarczak, P.) 381–409 (Instytut Archeologii i Etnologii Polskiej Akademii Nauk, 2016).

42. Zakościelna, A. & Taras, H. Badania ratownicze cmentarzysk kultury strzyżowskiej na Grzędzie Horodelskiej. *Archeologia Polski Środkowowschodniej* **1**, 49–57 (1996).

43. Kozak-Zychman, W. & Tajer, A. Analiza materiałów kostnych z grobów kultury strzyżowskiej na stanowisku 27 i 28 w Strzyżowie, woj. zamojskie. *Archeologia Polski Środkowowschodniej* **2**, 269–272 (1997).

44. Makarowicz, P. *Trzciniecki krąg kulturowy-wspólnota pograniczna Wschodu i Zachodu Europy*. (Wydawnictwo Poznańskie, 2010).

45. Kośko, A. & Klochko, V. Trzciniec" – Borderland of Early Bronze Civilizations of Eastern and Western Europe. *Baltic-Pontic Studies* **6**, 190–202 (1998).

46. Górski, J. The Trzciniec culture. On the periphery of Bronze Age civilization (1800-1100 BC). in *The Past Societies. Polish Lands from the first evidence of human presence to the Early Middle Ages. 3. 2000-500 BC* (eds. Urbańczyk, P. & Bugaj, U.) 87–126 (Instytut Archeologii i Etnologii Polskiej Akademii Nauk, 2017).

47. Makarowicz, P. Geneza pochówków zbiorowych w trzcinieckim kręgu kulturowym. in *Mente et rutro. Studia archaeologica Johanni Machnik viro doctissimo octogesimo vitae anno ab amicis, collegis et discipulis oblata* (eds. Czopek, S. & Kadrow, S.) 379–398 (Instytut Archeologii Uniwersytetu Rzeszowskiego, Fundacja Rzeszowskiego Ośrodka Archeologicznego, 2010).

48. Górski, J., Makarowicz, P. & Wawrusiewicz, A. *Osady i cmentarzyska społeczności trzcinieckiego kręgu kulturowego w Polesiu, stanowisko 1*. (Instytut Archeologii Uniwersytetu Łódzkiego : Fundacja Uniwersytetu Łódzkiego, 2011).

49. Górski, J. & Makarowicz, P. Nowe datowania radiowęglowe kurhanów i grobów beznasypowych trzcinieckiego kręgu kulturowego z Małopolski i Wielkopolski. in *Peregrinationes archaeologicae in Asia et Europa Joanni Chochorowski dedicatae* (ed. Blajer, W.) 289–298 (Instytut Archeologii Uniwersytetu Jagiellońskiego : Wydawnictwo Profil-Archeo, 2012).

50. Matoga, A. Wstępne wyniki badań na cmentarzysku z III-V okresu epoki brązu w Bocheńcu, woj. Kielce. *Spraw. Arch.* **27**, 81–109 (1985).

51. Szczepanek, A. Pochówki zbiorowe kultury trzcinieckiej z Bocheńca, stan. 2, woj. świętokrzyskie i Gustorzyna, stan. 1, woj. kujawsko-pomorskie – analiza antropologiczna. *Spraw. Arch.* **53**, 289–312 (2001).

52. Szczepanek, A. *Archeotanatologia pochówków zbiorowych od pradziejów po czasy współczesne*. (Wydawnictwo i Drukarnia MITEL, 2013).

53. Matoga, A. Problem przynależności kulturowej grobów szkieletowych z Bocheńca, woj. kieleckie. in *Kultura trzciniecka w Polsce* (eds. Polska, P. & Rydzewski, J.) 119–131 (Akademia Górniczo-Hutnicza im. S. Staszica, 1987).

54. Padło, K. & Ratajczak, R. *Brodzica, stan. 19, AZP 87-93/34. Przedinwestycyjne badania archeologiczne na trasie budowy gazociągu wysokiego ciśnienia z Moroczyna do Werbkowic w powiecie hrubieszowskim.* („SUB TERRA" Badania Archeologiczne, 2007).

55. Banasiewicz-Szykuła, E., Gołub, I., Koman, W., Stachyra, A. & Wetoszka, B. Sprawozdanie z działalności w zakresie ochrony zabytków archeologicznych na terenie woj. lubelskiego w 2007 roku. *Wiadomości Konserwatorskie Województwa Lubelskiego* **10**, 25–69.

56. Padło, K., Ratajczak, R. & Zakościelna, A. Nowe materiały kultury malickiej ze wschodniej Lubelszczyzny. in *Hereditas praeteriti: additamenta archaeologica et histica dedicata Ioanni Gurba*

Octogesimo Anno Nascendi H. Taras and A. Zakościelna (Eds.) 105–111 (Wydawnictwo Uniwersytetu Marii Curie-Skłodowskiej, 2009).

57. Florek, M. & Taras, H. *Dacharzów : cmentarzysko kultury trzcinieckiej*. (Uniwersytet Marii Curie-Skłodowskiej, 2003).

58. Górski, J. Cmentarzysko kultury trzcinieckiej w Guciowie, stan. 6, pow. zamojski. in *Fenomen miejsca : nekropola kurhanowa z neolitu, epoki brązu i wczesnego średniowiecza w Guciowie, pow. zamojski* (eds. Górski, J. & Tyniec, A.) 87–189 (Muzeum Archeologiczne w Krakowie, 2018).

59. Grygiel, R. & Pelisiak, A. Gustorzyn, woj., włocławskie. Stanowisko 1. *Informator Archeologiczny* 38–39 (1982).

60. Grygiel, R. Z badań nad kulturą trzciniecką w rejonie Brześcia Kujawskiego. in *Kultura trzciniecka w Polsce* (eds. Polska, P. & Rydzewski, J.) 73–88 (Akademia Górniczo-Hutnicza im. S. Staszica, 1987).

61. Pospieszny, Ł. *et al.* Isotopic evidence of millet consumption in the Middle Bronze Age of East-Central Europe. *Journal of Archaeological Science* **126**, 105292 (2021).

62. Makarowicz, P. The birth of a new world. Barrows, warriors, and metallurgists. in *The Past societies. Polish lands from the first evidence of human presence to the early Middle Ages. 3. 2000-500 BC* (ed. Bugaj, U.) 127–186 (Instytut Archeologii i Etnologii Polskiej Akademii Nauk, 2017).

63. Wójcik, I. & Szmoniewski, B. Śmietnisko muszlowe kultury trzcinieckiej w Koszycach, pow. proszowicki. *Materiały Archeologiczne* **41**, 169–181 (2016).

64. Górski, J. Pochówki zwierząt i depozyty zwierzęce w kulturze trzcinieckiej (wstęp do problematyki). in *Na pograniczu światów. Studia z pradziejów międzymorza bałtycko-pontyjskiego ofiarowane Profesorowi Aleksandrowi Kośko w 60. rocznicę urodzin.* (eds. Bednarczyk, J., Czebreszuk, J., Makarowicz, P. & Szmyt, M.) 105–114 (Wydawnictwo Poznańskie, 2008).

65. Górski, J. & Makarowicz, P. Human remains of the Trzciniec circle in the settlement contexts: extraordinary burials or offerings? in *Space not only for the living: Human remains at Bronze Age settlements in Eurasia.* (eds. Kaiser, E. & Berseneva, N.) (in press, 2023).

66. Rachwaniec, A. Materiały archeologiczne ze starszego okresu epoki brązu oraz kultury łużyckiej z rejonu Kopca Wandy w Nowej Hucie-Mogile. *Materiały Archeologiczne Nowej Huty* **9**, 89–191 (1985).

67. Górski, J. Osada kultury trzcinieckiej i łużyckiej w Nowej Hucie-Mogile, stanowisko 55. Analiza materiałów część I. *Materiały Archeologiczne Nowej Huty* **16**, 55–102 (1993).

68. Górski, J. Osada kultury trzcinieckiej i łużyckiej w Nowej Hucie-Mogile, stanowisko 55. Analiza materiałów część II. *Materiały Archeologiczne Nowej Huty* **17**, 65–113 (1994).

69. Górski, J. & Wróbel, J. Zbiorowy grób szkieletowy kultury trzcinieckiej z Nowej Huty-Cła, stan. 65. *Materiały Archeologiczne Nowej Huty* **22**, 21–34 (2000).

70. Przybyła, M., Tunia, K. & Szczepanek, A. Grób kultury trzcinieckiej w południowej części stanowiska 5 (G) w Słonowicach, pow. Kazimierza Wielka. in *Vir Bimaris : od kujawskiego matecznika do stepów nadczarnomorskich : studia z dziejów międzymorza bałtycko-pontyjskiego ofiarowane profesorowi Aleksandrowi Kośko* (eds. Szmyt, M., Chachlikowski, P., Czebreszuk, J., Ignaczak, M. & Makarowicz, P.) 435–445 (Instytut Archeologii Uniwersytetu im. Adama Mickiewicza w Poznaniu, 2019).

71. Włodarczak, P. Groby kultury mierzanowickiej oraz kultury trzcinieckiej z Żernik Górnych. in *'Trzciniec' : system kulturowy czy interkulturowy proces?* (eds. Kośko, A. & Czebreszuk, J.) 161–177 (Wydawnictwo Poznańskie, 1998).

72. Bieriezanska, Z. Osada kultury wschodniotrzcinieckiej nad Dnieprem. *Z Otchłani Wieków* **33**, 205–211 (1967).

73. Swiesznikow, I. K. Kultura komarowska. *Archeologia Polski* **12**, 39–107 (1967).

74. Sveshnikov, I. K. Srednij period bronzovogo veka Prikarpat'ja i Volyni. Tshinetsko-komarovskaja kultura. in *Arkheologija Prikarpat'ja, Volyni i Zakarpatja* (ed. Chernysh, O. P.) 78–88 (Nauk. Dumka, 1990).

75. Dąbrowski, J. *Powiązania ziem polskich z terenami wschodnimi w epoce brązu*. (Zakład Narodowy im.Ossolińskich, 1972).

76. Romaniszyn, J., Niculică, B. P. & Ignat, I. Funeral rites on the southern boundary of the Komarov culture. *Spraw. Arch.* **69**, 83–112 (2017).

77. Makarowicz, P. Groby z II tys. przed Chr. Nekropola kultury komarowskiej nad Seretem/ Graves from the 2nd mill. BC. A Komarów culture necropolis by the river Seret. in *Biały Potok : materiały z badań Józefa Kostrzewskiego na Podolu* (ed. Szmyt, M.) 455–484 (Muzeum Archeologiczne w Poznaniu, 2016).

78. Romaniszyn, J. & Makarowicz, P. Real Burials or Cenotaphs? A Study of the Mysterious Under-mound Funerary Constructions of the Trzciniec Cultural Circle. *Archaeology and Early History of Ukraine* **39**, 360–374 (2021).

79. Romaniszyn, J. & Makarowicz, P. The Biały Potok group as an enigmatic problem in the first half of the 2nd millennium BC in West Podolia. The current problem of interpretation. *Fontes Archaeologici Posnanienses* **54**, 31–55 (2018).

80. Sulimirski, T. *Corded ware and globular amphorae north-east of the Carpathians*. (Athlone, 1968).

81. Kirkor, A. Sprawozdanie i wykaz zabytków złożonych w Akademii Umiejętności z wycieczki archeologiczno-antropologicznej w roku 1877. *Zbiór Wiadomości do Antropologii Krajowej* **2**, 3–18 (1878).

82. Romaniszyn, J., Gwizdała, M. & Makarowicz, P. Grób z Beremian nad Dniestrem – rewizja wyników badań z XIX wieku. Ze studiów nad grupą białopotocka kultury komarowskiej. *Materiały Archeologiczne* **41**, 7–21 (2016).

83. Kostrzewski, J. Groby eneolityczne z skurczonemi szkieletami w Białym Potoku (w pow. czortkowskim). *Przegląd Archeologiczny* **3**, 9–17 (1926).

84. Makarowicz, P., Szmyt, M. & Popa, C. I. Biały Potok Cemetery Revisited. in *The Carpathian Basin and the Northern Balkans between 3500 and 2500 BC : common aspects and regional differences* 233–261 (Editura Mega, 2016).

85. Ilchyshyn, V. Komarivskiy gorizont bagatoscharovogo kurgannogo mogilnika Kordishiv VIII na Schumshchini. *Materialy i Doslidžennja z Archeolohii Prykarpattja i Volyni* **20**, 259–272 (2016).
