# Supplementary material for: Patrilocality and hunter-gatherer-related ancestry of populations in East-Central Europe during the Middle Bronze Age
Source: Nat Commun. 2023 Aug 1;14:4395. doi: 10.1038/s41467-023-40072-9 (PMC10393988; doi:10.1038/s41467-023-40072-9)
Supplement: Supplementary file 2 — Description of Additional Supplementary Files [file 41467_2023_40072_MOESM2_ESM.pdf]

Description of Additional Supplementary Files

File Name: Supplementary Data 1
Description: The archaeological context and basic sequencing statistics for all the individuals sampled for the project.

File Name: Supplementary Data 2
Description: The archaeological context, summary of some results and labels used in downstream analysis, for the individuals selected for the study (passing quality and coverage thresholds).

File Name: Supplementary Data 3
Description: Ancient reference individuals used in the study.

File Name: Supplementary Data 4
Description: Results of calling genotypes for 69 391 no-indel branch determining Y SNPs obtained from the International Society of Genetic Genealogy collection.

File Name: Supplementary Data 5
Description: The results of population vs population f3 statistics on autosomes.

File Name: Supplementary Data 6
Description: The results of population vs population f3 statistics on chromosome X.

File Name: Supplementary Data 7
Description: The results of individual vs population f3 statistics on autosomes.

File Name: Supplementary Data 8
Description: The results of individual vs population f3 statistics on chromosome X.

File Name: Supplementary Data 9
Description: The results of pairwise, individual vs individual f3 statistics on autosomal data.

File Name: Supplementary Data 10
Description: The results of D statistics in a form: D(YRI, Żerniki individual, Żerniki population, other TC individuals)

File Name: Supplementary Data 11
Description: Standard error values of the cross-validation error estimate for Admixture analysis.

File Name: Supplementary Data 12
Description: The results of D statistics in a form: D(YRI, ancient individual, population1, population2)

File Name: Supplementary Data 13
Description: The results of qpAdm analysis for 2-way admixture models.

File Name: Supplementary Data 14
Description: The results of qpAdm analysis for 3-way admixture models.

File Name: Supplementary Data 15
Description: The results of qpAdm analysis for 2-way admixture models, using rotating outgroup approach.

File Name: Supplementary Data 16
Description: The results of qpAdm analysis for 3-way admixture models, using rotating outgroup approach.

File Name: Supplementary Data 17
Description: The results of qpAdm analysis for 3-way individual level admixture models.

File Name: Supplementary Data 18
Description: The results of NGSrelate kinship analysis on autosomal data.

File Name: Supplementary Data 19
Description: The results of NGSrelate kinship analysis on X chromosome.

File Name: Supplementary Data 20
Description: The results of READ kinship analysis on autosomal data.

File Name: Supplementary Data 21
Description: The summary of the kinship analyses with final interpretations.
