# Supplementary material for: Patrilocality and hunter-gatherer-related ancestry of populations in East-Central Europe during the Middle Bronze Age
Source: Nat Commun. 2023 Aug 1;14:4395. doi: 10.1038/s41467-023-40072-9 (PMC10393988; doi:10.1038/s41467-023-40072-9)
Supplement: Supplementary file 4 — Reporting Summary [file 41467_2023_40072_MOESM4_ESM.pdf]

nature portfolio

Corresponding author(s): Maciej Chyleński
Anna Juras
Helena Malmström

Last updated by author(s): Jun 2, 2023

Reporting Summary

Nature Portfolio wishes to improve the reproducibility of the work that we publish. This form provides structure for consistency and transparency in reporting. For further information on Nature Portfolio policies, see our Editorial Policies and the Editorial Policy Checklist.

Statistics

For all statistical analyses, confirm that the following items are present in the figure legend, table legend, main text, or Methods section.

n/a	Confirmed	
☐	☒	The exact sample size (*n*) for each experimental group/condition, given as a discrete number and unit of measurement
☐	☒	A statement on whether measurements were taken from distinct samples or whether the same sample was measured repeatedly
☐	☒	The statistical test(s) used AND whether they are one- or two-sided<br>*Only common tests should be described solely by name; describe more complex techniques in the Methods section.*
☐	☒	A description of all covariates tested
☒	☐	A description of any assumptions or corrections, such as tests of normality and adjustment for multiple comparisons
☐	☒	A full description of the statistical parameters including central tendency (e.g. means) or other basic estimates (e.g. regression coefficient) AND variation (e.g. standard deviation) or associated estimates of uncertainty (e.g. confidence intervals)
☐	☒	For null hypothesis testing, the test statistic (e.g. *F*, *t*, *r*) with confidence intervals, effect sizes, degrees of freedom and *P* value noted<br>*Give P values as exact values whenever suitable.*
☒	☐	For Bayesian analysis, information on the choice of priors and Markov chain Monte Carlo settings
☒	☐	For hierarchical and complex designs, identification of the appropriate level for tests and full reporting of outcomes
☒	☐	Estimates of effect sizes (e.g. Cohen's *d*, Pearson's *r*), indicating how they were calculated

Our web collection on statistics for biologists contains articles on many of the points above.

Software and code

Policy information about availability of computer code

Data collection	no software was used for data colection
Data analysis	AdapterRemoval v 2.1.7, MergeReadsFastQ_cc.py, Burrows-Wheeler Aligner v 0.7.17-r1188, FilterUniqueSAMCons.py, samtools v1.5, MapDamage v 2.0.8, ContamMix v 1.0.10, ANGSD v 0.910, Haplogrep (v 2.4.0), PhyloTree (phylogenetic tree build 17), aDNA_GenoCaller.py, vcftools (v 0.1.16), READ, NgsRelate v 2.0, EIGENSOFT (v 7.2.1), ADMIXTURE (v 1.3), PLINK (v 1.90b5), admixtools v 5.1, R v 4.2.2

For manuscripts utilizing custom algorithms or software that are central to the research but not yet described in published literature, software must be made available to editors and reviewers. We strongly encourage code deposition in a community repository (e.g. GitHub). See the Nature Portfolio guidelines for submitting code & software for further information.

Data

Policy information about availability of data

All manuscripts must include a data availability statement. This statement should provide the following information, where applicable:

- Accession codes, unique identifiers, or web links for publicly available datasets
- A description of any restrictions on data availability
- For clinical datasets or third party data, please ensure that the statement adheres to our policy

Datasets used in the study: human reference genome assembly GRCh37, Human Origins and Simons Genome Diversity Project Datasets (https://reich.hms.harvard.edu/datasets), 1000 genome dataset (https://ftp.1000genomes.ebi.ac.uk/). Ancient reference genomes deposited in European Nucleotide

Archive under accession numbers: PRJEB11364, PRJEB12155, PRJEB13123, PRJEB14180, PRJEB14455, PRJEB20658, PRJEB21037, PRJEB21940, PRJEB22592, PRJEB22629, PRJEB22652, PRJEB22652, PRJEB23635, PRJEB26349, PRJEB27628, PRJEB28451, PRJEB29074, PRJEB29603, PRJEB29700, PRJEB26336., PRJEB31035, PRJEB32466, PRJEB32786, PRJEB34091, PRJEB34400, PRJEB36854, PRJEB6090, PRJEB6272, PRJEB8987, PRJEB9021, PRJNA295861 and PRJNA318237. Additional ancient genomic data from Sequence Read Archive deposited under the accession numbers SAMN08139261–SAMN08139301, SAMN14206592–SAMN14206687 and SRP029640.
The aligned sequencing data have been deposited in the European Nucleotide Archive database (https://www.ebi.ac.uk/ena/browser/home) under accession number (PRJEB53670).

Research involving human participants, their data, or biological material

Policy information about studies with human participants or human data. See also policy information about sex, gender (identity/presentation), and sexual orientation and race, ethnicity and racism.

Reporting on sex and gender	not applicable
Reporting on race, ethnicity, or other socially relevant groupings	not applicable
Population characteristics	not applicable
Recruitment	not applicable
Ethics oversight	not applicable

Note that full information on the approval of the study protocol must also be provided in the manuscript.

Field-specific reporting

Please select the one below that is the best fit for your research. If you are not sure, read the appropriate sections before making your selection.

☒ Life sciences ☐ Behavioural & social sciences ☐ Ecological, evolutionary & environmental sciences

For a reference copy of the document with all sections, see nature.com/documents/nr-reporting-summary-flat.pdf

Life sciences study design

All studies must disclose on these points even when the disclosure is negative.

Sample size	The data was collected from available skeletal remains deposited in various academic and museal Institutions. In total 175 samples were collected. No selection process was implemented, all available samples (based on literature overview and our knowledge) for which appropriate permits were given, were collected.
Data exclusions	82 samples were excluded due to insufficient DNA preservation, and one sample was excluded to due to possible contamination with modern DNA.
Replication	Replication in aDNA studies is regularly performed in certain analysis (such as Admixture) and implemented in the used software. Various approaches are used depending of the tool and the detailed information can be found in the manuals and publications describing each of them. Additionally, the conclusion drawn in the article are based on number of different methods and usually at least two different tools are used to address one research question. All attempts at replication were successfu
Randomization	Due to small sample size, characteristic for aDNA studies, no randomization when choosing samples for analysis was implemented. Where possible analysis was performed for each individual separately. The samples were grouped according to their age, region of origin and archaeological context. In cases in which individual samples did not cluster together with other individuals belonging to the same category, in primary PCA analysis. they were treated as an outlier and removed from further population level analyses.
Blinding	Blinding is uncommon in aDNA studies as the amount of available data is small. However, upon arrival to aDNA laboratory, the samples were given undescriptive lab codes, and matched with their context only during the data analysis.

Reporting for specific materials, systems and methods

We require information from authors about some types of materials, experimental systems and methods used in many studies. Here, indicate whether each material, system or method listed is relevant to your study. If you are not sure if a list item applies to your research, read the appropriate section before selecting a response.

Materials & experimental systems

n/a	Involved in the study
☒	☐ Antibodies
☒	☐ Eukaryotic cell lines
☐	☒ Palaeontology and archaeology
☒	☐ Animals and other organisms
☒	☐ Clinical data
☒	☐ Dual use research of concern
☒	☐ Plants

Methods

n/a	Involved in the study
☒	☐ ChIP-seq
☒	☐ Flow cytometry
☒	☐ MRI-based neuroimaging

Palaeontology and Archaeology

Specimen provenance

The detailed information on the provenance of each sample can be found in the Supplementary text file attached to the manuscript. For each sample appropriate permit was acquired from the institution providing the access to the specimen, and the sample providers coauthored the manuscript:
The access to human remains from sites: Beremiany, Bocheniec, Guciów, Koszyce, Kraków Nowa Huta, Kopiec Wandy, Pieczeniegi, Pielgrzymowice, Świniary Stare and Wyciąż was granted in 2015 by Jacek Górski representing the Archaeological Museum in Cracow.
The access to human remains from Biały Potok was granted in 2015 by Marzena Szmyt, head of Archaeological Museum in Poznań.
The materials from sites: Brodzica, Hrebenne, Nieledew and Raciborowice were sampled following the permision granted in 2016 by Marta Polańska representing the Department of Material and Spiritual Culture, Lublin Museum.
The samples from: Dacharzów, Strzyżów and Zubowice were taken following the permission granted in 2016 by Halina Taras representing the Institute of Archaeology, Maria Curie-Skłodowska University.
The human remains from the sites in Łojewo, Piecki, Siniarzewo and Karczyn were accessed in 2016 based on the agreement with Przemysław Makarowicz representing the Faculty of Archaeology, Adam Mickiewicz University in Poznan.
The specimens from sites in Stryjów, Słonowice and Żerniki Górne were sampled in 2017 following an agreement with Piotr Włodarczak representing Institute of Archaeology and Ethnology, Polish Academy of Science.
The specimen from Gąsawa was sampled based on the agreement from 2016 with Anna Grossmann representing the archaeological Museum in Biskupin.
Human remains from Gustorzyn site were accessed based on the permission granted in 2018 by Ryszard Grygiel, director of Museum of Archaeology and Ethnography in Łódź.
The samples from Kordashiv were acquired in 2020 based on the permission granted by Vasyl Ilchyshyn representing the Zaliztsi Museum of Local Lore.
The access to Mokrzec individual was granted in 2019 by Krzysztof Szostek representing Institute of Biological Sciences, Cardinal Stefan Wyszynski University in Warsaw.

Specimen deposition

Whole specimens were not collected, only small samples from each individual. The remaining skeletal materials are deposited in respective academic and museal institutions.

Dating methods

No new dates are provided.

☐ Tick this box to confirm that the raw and calibrated dates are available in the paper or in Supplementary Information.

Ethics oversight

No Ethical approval or guidance is required by Polish or Ukrainian law, or by any of our Funding providers for studying prehistoric human remains.

Note that full information on the approval of the study protocol must also be provided in the manuscript.
